# NAT10 Increases Lysosomal Acidification to Promote Esophageal Cancer Metastasis via ac4C Acetylation of ATP6V0E1 mRNA

**DOI:** 10.1002/advs.202502931

**Published:** 2025-07-29

**Authors:** Yu‐Juan Zhan, Chun‐Miao Deng, Lin Tang, Shu‐Jun Li, Tao‐Yang Xu, Xian Wei, Xin‐Yi Zhang, Can‐Can Zheng, Li Deng, Cui Shao, Zhong‐Min Ouyang, Alfred King‐Yin Lam, Rong Zhang, Jun Liu, Xing‐Yuan Shi, Zhen‐Yu Pan, Wei Dai, Ming‐Liang He, Simon Law, Xu Li, Xiao‐Bing Chen, Cheng Zhou, Bin Li, Wen‐Wen Xu

**Affiliations:** ^1^ State Key Laboratory of Respiratory Disease Key Laboratory of Biological Targeting Diagnosis Therapy and Rehabilitation of Guangdong Higher Education Institutes The Fifth Affiliated Hospital of Guangzhou Medical University Guangzhou 510700 China; ^2^ State Key Laboratory of Respiratory Disease Guangdong Provincial Key Laboratory of Protein Modification and Degradation School of Basic Medical Sciences The Affiliated Traditional Chinese Medicine Hospital Guangzhou Medical University Guangzhou 511400 China; ^3^ The Affiliated Traditional Chinese Medicine Hospital Sleep Research Institute of Traditional Chinese Medicine Guangzhou Medical University Guangzhou 510000 China; ^4^ Department of Radiology The Fifth Affiliated Hospital of Guangzhou Medical University Guangzhou 510700 China; ^5^ Cancer Molecular Pathology and Griffith Medical School Griffith University Gold Coast Queensland 4222 Australia; ^6^ Department of Endoscopy State Key Laboratory of Oncology in South China Guangdong Provincial Clinical Research Center for Cancer Sun Yat‐sen University Cancer Center Guangzhou 510060 China; ^7^ State Key Laboratory of Respiratory Disease and National Clinical Research Center for Respiratory Disease Department of Thoracic Surgery and Oncology The First Affiliated Hospital of Guangzhou Medical University Guangzhou 510030 China; ^8^ Department of Radiation Oncology The Fifth Affiliated Hospital of Guangzhou Medical University Guangzhou 510700 China; ^9^ Department of Radiation Oncology The Affiliated Huizhou Hospital Guangzhou Medical University Huizhou 516000 China; ^10^ Department of Clinical Oncology Li Ka Shing Faculty of Medicine The University of Hong Kong Hong Kong 999077 China; ^11^ Department of Biomedical Sciences City University of Hong Kong Hong Kong 518057 China; ^12^ Department of Thoracic Surgery First Affiliated Hospital of Fujian Medical University Fuzhou Fujian 350000 China; ^13^ Department of Oncology Henan Engineering Research Center of Precision Therapy of Gastrointestinal Cancer & Zhengzhou Key Laboratory for Precision Therapy of Gastrointestinal Cancer The Affiliated Cancer Hospital of Zhengzhou University & Henan Cancer Hospital Zhengzhou 450000 China; ^14^ Department of Radiation Oncology Nanfang Hospital Southern Medical University Guangzhou 510515 China

**Keywords:** ac4C modification, esophageal cancer metastasis, lysosomal acidification, NAT10, targeted therapy

## Abstract

N‐acetyltransferase 10 (NAT10)‐catalyzed N4‐acetylcytidine (ac4C) modification has been reported to drive tumor metastasis. Lysosomal dysregulation plays an important role in human diseases, but its function in esophageal cancer metastasis is unclear. It remains unknown whether NAT10 regulates lysosomal function, and the underlying mechanism and treatment strategy warrants investigation. Here, a novel role of NAT10 in inducing lysosomal acidification is revealed, and the clinical and biological significance of ATP6V0E1 in tumor metastasis is uncovered. Mechanistically, NAT10 promotes the translation efficiency of ATPase H^+^ transporting V0 subunit e1 (ATP6V0E1) mRNA in an ac4C‐dependent manner to facilitate ATP6V0E1 expression and vacuolar H^+^‐ATPase (v‐ATPase) activity, enhancing the lysosomal degradation of E‐cadherin, ultimately accelerating tumor metastasis. Furthermore, G‐749 is screened and identified as a novel NAT10 inhibitor capable of effectively impeding lysosomal acidification and tumor metastasis by disrupting the NAT10‐Ubiquitin‐specific Peptidase 39 (USP39) interaction. Overall, the results unveil a novel role of ac4C modifications in regulating lysosomal acidification and propose a potential strategy by targeting NAT10 to inhibit esophageal cancer metastasis.

## Graphical abstract

Zhan et al. demonstrate the vital role of NAT10‐catalyzed ac4C modification in regulating autophagy‐associated lysosomal acidification. They uncover the clinical and biological significance of ATP6V0E1, an important v‐ATPase assembly subunit, in tumor metastasis, particularly in the malignant progression of ESCC. Furthermore, they identify G‐749 as a potential NAT10 inhibitor to suppress cancer metastasis by impairing lysosomal acidification.

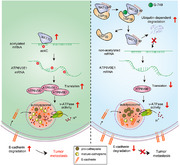

## 1 Introduction

Metastasis is the most prominent hallmark of human cancer and significantly contributes to mortality.^[^ ^1^ ^]^ There is an urgent need to explore therapeutic biomarkers and develop treatment strategies for cancer metastasis. It is gaining increasing recognition that the underlying mechanisms driving cancer progression cannot be entirely explained by genomic alterations.^[^ ^2^ ^]^ RNA epitranscriptomics, in particular, RNA modifications, have been demonstrated to participate in multiple biological processes of human diseases.^[^ ^3^ ^]^ Recently, N4‐acetylcytidine (ac4C) acetylation modification has been discovered on mammalian mRNA, enhancing both mRNA stability and translation efficiency, with N‐acetyltransferase 10 (NAT10) as the only known modification enzyme.^[^ ^4^ ^]^ Interestingly, NAT10, as the only protein with an acetylase structural domain and an RNA‐binding structural domain simultaneously, has recently been reported to promote tumorigenesis and tumor metastasis,^[^ ^4^, ^5^ ^]^ but its action mechanisms remain poorly elucidated.

Autophagy, a fundamental cellular process that eliminates molecules and subcellular elements to balance sources of energy, has been suggested to be involved in modulating tumor cell motility and invasion.^[^ ^6^ ^]^ Autophagy initiates with the formation of double membranes that engulf cytoplasmic cargoes and extend to form autophagosomes, which eventually fuse with lysosomes to generate autolysosomes.^[^ ^7^ ^]^ Thus, autophagy largely depends on the acidic environment within lysosomes, which is maintained by the proton‐pump activity of vacuolar H^+^‐ATPase (v‐ATPase)^[^ ^8^ ^]^ and played an important role in tumor metastasis.^[^ ^9^ ^]^ V‐ATPase is a multisubunit complex consisting of a peripheral V1 domain and a transmembrane V0 domain.^[^ ^10^ ^]^ Up to now, it remains unclear whether NAT10‐mediated ac4C modification regulates v‐ATPase subunits and the autophagy‐lysosomal pathway. Here, through the integration of acetylated RNA immunoprecipitation and sequencing (acRIP‐seq) and a series of experiments, only ATPase H^+^ transporting V0 subunit e1 (ATP6V0E1) was found to undergo ac4C modification among v‐ATPase‐related subunits. As an important component of v‐ATPase, a proton‐pump in the lysosomal membrane, ATP6V0E1 is responsible for regulating intracavity acidity.^[^ ^10^, ^11^ ^]^ Till now, the biological function and clinical significance of ATP6V0E1 in tumorigenesis have not been reported.

As the seventh most common and sixth most deadly cancer globally, esophageal cancer is a key threat to human health. Esophageal squamous cell carcinoma (ESCC) accounts for more than 70% of esophageal cancers and is related to extensive invasion and metastasis.^[^ ^12^ ^]^ However, there is a scarcity of targeted drugs for this lethal disease and it is of great urgency to develop an effective strategy. In this study, we aim to study the important role of NAT10‐mediated ac4C modification in regulating lysosomal acidification. The biological function of ATP6V0E1 as a novel substrate of ac4C modification to promote cancer metastasis and its clinical significance in ESCC were investigated. Our data also suggest that selectively targeting ac4C modification to inhibit lysosomal dysregulation may present a potential therapeutic strategy. Therefore, in this study, we aimed to elucidate a novel mechanism underlying tumor metastasis and provide potential treatment strategy for the patients with advanced ESCC.

## 2 Results

### 2.1 NAT10 Enhances the Lysosomal Acidification Through Increasing v‐ATPase Activity

**Figure 1:**
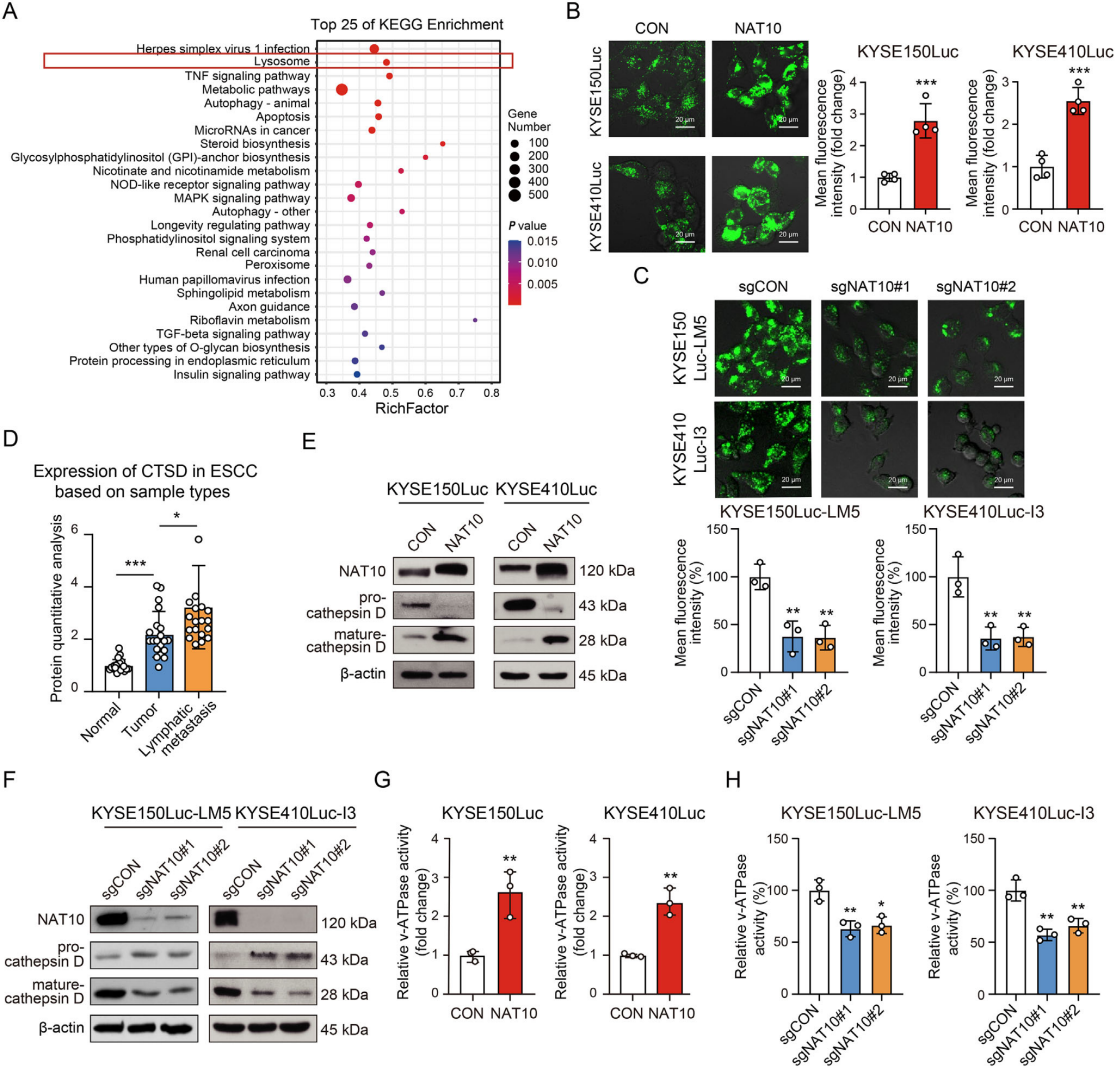
NAT10 enhances the lysosomal acidification through increasing v‐ATPase activity. A) KEGG pathway enrichment analysis was conducted on significantly differentially expressed genes in cells overexpressing NAT10 or control. B,C) LysoTracker‐Green staining was used to evaluate the lysosomal acidification status in KYSE150Luc and KYSE410Luc cells upon NAT10 overexpression or control (B), and KYSE150Luc‐LM5 and KYSE410Luc‐I3 cells with NAT10 knockout (C). Scale bar: 20 µm. D) Expression of Cathepsin D (CTSD) among primary ESCC tumor tissues (Tumor), matched normal tissues (Normal), and lymph node metastatic tissues (Lymphatic metastasis) (*n* = 20). E,F) Western blot analysis showing expression of pro‐cathepsin D and mature‐cathepsin D in NAT10‐expressing KYSE150Luc and KYSE410Luc cells or control (E), as well as in NAT10‐knockout KYSE150Luc‐LM5 and KYSE410Luc‐I3 cells (F). G,H) Detection of v‐ATPase activity in KYSE150Luc and KYSE410Luc cells with NAT10 overexpression (G), or KYSE150Luc‐LM5 and KYSE410Luc‐I3 cells with NAT10 knockout (H). * *P* < 0.05; ** *P* < 0.01; *** *P* < 0.001.

Although NAT10 has been reported to promote the invasion of ESCC cells in our previous study,^[^ ^5^ ^]^ the underlying molecular mechanism remains to be investigated (Figure S1A,B, Supporting Information). Analysis of transcriptome sequencing (RNA‐Seq) data identified ≈5929 differentially expressed genes upon NAT10 overexpression (log_2_Fold Change > 2.0), including 4420 up‐regulated genes and 1509 down‐regulated genes. A subsequent Kyoto Encyclopedia of Genes and Genomes (KEGG) pathway enrichment analysis indicated that the lysosomal pathway ranked top, except for the herpes simplex virus‐1 infection pathway (**Figure** **1**A). Based on 5 up‐regulated and 5 down‐regulated lysosomal‐related genes in NAT10‐overexpressing cells from KEGG analysis, we further performed the Reverse Transcription Quantitative Polymerase Chain Reaction (RT‐qPCR) analysis and got the consistent findings (Figure S1C, Supporting Information). Therefore, lysosomal function became the focus of this study for exploring the biological role of ac4C modification. Lysosomes require an acidic environment to initiate proteolysis and support the progression of autophagy.^[^ ^13^ ^]^ An acid‐dependent green fluorescent dye, LysoTracker, was used to assess lysosomal pH, and the results showed that overexpression of NAT10 resulted in a significant increase in lysosomal acidity, whereas knockout of NAT10 decreased lysosomal acidity (Figure 1B,C). Notably, cathepsins A, B, C, D, H, L, S, and Z are representative hydrolytic enzymes influenced by an acidic environment.^[^ ^14^ ^]^ Our data from a systematic proteomic analysis of 20 cases of primary ESCC tumor tissues, matched normal tissues, and lymph node metastatic tissues revealed that the expression of cathepsins A, B, C, D, H, L, S, and Z were elevated in tumor tissues (Figure 1D; Figure S1D, Supporting Information). Among them, cathepsins D, H, L, S, and Z were further significantly upregulated in metastatic tissues, indicating a crucial role of lysosomal acidification in esophageal cancer metastasis. Next, we examined the effect of NAT10 on the conversion of cathepsin D, one of the most representative hydrolytic enzymes, from precursor to mature form, which is required for lysosomal function in decomposing misfolded proteins and damaged organelles.^[^ ^8^, ^13^, ^15^ ^]^ Interestingly, overexpression of NAT10 led to a notable increase in the maturation of cathepsin D in ESCC cells (Figure 1E). On the contrary, we observed the opposite results in the NAT10‐deficient cells (Figure 1F). These data highlight a novel function of NAT10 in increasing lysosomal acidification. Lysosomes maintain their pH gradient by pumping protons through regulating v‐ATPase activity. This process uses metabolic energy in the form of ATP to transport protons into the lysosomal lumen.^[^ ^13^ ^]^ The results revealed that overexpression of NAT10 led to an augmentation of v‐ATPase activity (Figure 1G), while the opposite result was obtained in the NAT10‐deficient cells (Figure 1H). Moreover, we found that overexpression of NAT10 induced a decrease in the expression of LC3‐II and p62 (Figure S2A, Supporting Information), while NAT10 knockout resulted in the simultaneous accumulation of LC3‐II and p62 (Figure S2B, Supporting Information), supporting the idea that NAT10 regulates the late stage of autophagic flux. We also observed that chloroquine abolishes the promoting effect of NAT10 on the invasion of ESCC cells (Figure S2C, Supporting Information). Therefore, we speculated that NAT10‐modulated lysosomal acidification promotes tumor metastasis through autophagy. The above data supported the vital biological function of NAT10 in causing excessive acidification by regulating ATP‐dependent proton pump activities.

### 2.2 NAT10‐Catalyzed ac4C Modification Increases the Translation Efficiency of ATP6V0E1 mRNA

**Figure 2:**
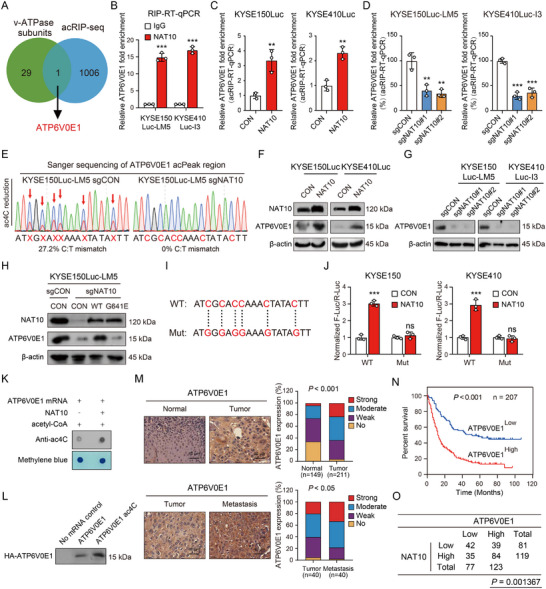
NAT10‐catalyzed ac4C modification increases the translation efficiency of ATP6V0E1 mRNA. A) Diagram showing the strategy to screen target genes via overlapping the v‐ATPase subunits and acRIP‐seq gene lists. B) RIP‐RT‐qPCR was applied to detect the binding between NAT10 and ATP6V0El mRNA using NAT10 antibody or normal mouse IgG control in KYSE150Luc‐LM5 and KYSE410Luc‐13 cells. C,D) acRIP‐RT‐qPCR was performed to detect the ac4C modification level of ATP6V0E1 mRNA in KYSE150Luc and KYSE410Luc cells with NAT10‐expression (C) and KYSE150Luc‐LM5 and KYSE410Luc‐I3 cells with NAT10‐knockout (D). E) Base resolution mapping of ATP6V0E1 in the acPeak region from Sanger sequencing (RedaC:T‐seq). F,G) Western blot analysis was performed to analyze ATP6V0E1 expression as indicated. H) Western blot showing the expression of ATP6V0E1 when transfected with wild‐type (WT) or mutant NAT10 (G641E). I) Mutants design of ATP6V0E1 acPeak. J) Translation efficiency of ATP6V0E1 was detected in ESCC cells co‐transfected with plasmids expressing WT or mutant ATP6V0E1 acPeak and NAT10 using a luciferase reporter assay. K) Dot blot analysis determined the ac4C modification of ATP6V0E1 catalyzed by NAT10 in vitro. L) Western blot analysis assessed the in vitro translation efficiency of unmodified or ac4C‐modified ATP6V0E1 catalyzed by NAT10. M) Representative images and expression patterns of ATP6V0E1 in primary ESCC tumor tissues and adjacent normal tissues (upper panel), as well as in matched primary and metastatic tissues (lower panel). Scale bar: 40 µm. N) Kaplan‐Meier analysis evaluating the overall survival of ESCC patients according to ATP6V0E1 expression. O) Correlation analysis between the expression of NAT10 and ATP6V0E1. Bars, SDs; ns, no significance; ** *P* < 0.01; *** *P* < 0.001.

To explore the mechanism of how ac4C modification regulates v‐ATPase activity to increase lysosomal acidification, we analyzed acRIP‐seq data in NAT10‐overexpressing cells.^[^ ^5^ ^]^ Among all the v‐ATPase subunits (Table S1, Supporting Information), only ATP6V0E1, exhibited the upregulation of ac4C modification and became our research focus (**Figure** **2**A). Regarding ac4C modification in ATP6V0E1, according to the acRIP‐seq results, only one acPeak (Chromosome 5, thickStart: 173034766, thickEnd: 173034914) was significantly upregulated in NAT10‐overexpressing cells compared to control cells. To determine the binding between NAT10 and ATP6V0E1 mRNA, a RNA Immunoprecipitation (RIP) assay was performed using antibodies against NAT10, followed by RT‐qPCR using primers targeting the ATP6V0E1 acPeak region. The results confirmed that NAT10 binds directly to the ATP6V0E1 acPeak region (Figure 2B). Moreover, acRIP‐RT‐qPCR further confirmed that overexpression of NAT10 increased the ac4C modification levels of ATP6V0E1 mRNA, while the opposite results were observed in NAT10‐knockout cells (Figure 2C,D). To further verify the presence of the ac4C site in ATP6V0E1 mRNA, an orthogonal method with nucleotide resolution was employed. We found that ablation of NAT10 significantly decreased the C:T mismatch of the acPeak region in ATP6V0E1 mRNA compared to control cells, with an average reduction from 27.2% to 0%, validating the existence of ac4C modification in ATP6V0E1 mRNA (Figure 2E).

The positive regulatory effect of NAT10 on the expression of ATP6V0E1 protein was also confirmed (Figure 2F). NAT10‐G641 has been identified as a key amino acid for its acetyltransferase activity.^[^ ^4^, ^16^ ^]^ The results of Western blot showed that depletion of NAT10 resulted in a reduction in the protein expression of ATP6V0E1 (Figure 2G), which was mitigated when NAT10‐wild type (WT) was introduced, but not with the NAT10‐G641E mutation (Figure 2H). We hypothesized that ac4C modification might alter the stability of ATP6V0E1 mRNA. Unexpectedly, neither the overexpression nor the knockdown of NAT10 affected the half‐life of ATP6V0E1 mRNA (Figure S3A,B, Supporting Information). Next, luciferase reporter assay revealed that the translation efficiency of ATP6V0E1 mRNA with WT acPeak region was higher in cells overexpressing NAT10 compared to control cells (Figure 2I,J). However, this effect was not observed in the cells with the C to G mutant in the acPeak region. Furthermore, we performed in vitro transcription of ATP6V0E1 mRNA using T7 RNA polymerase, and acetylated the mRNA with NAT10 and acetyl‐CoA (Figure 2K). The in vitro translation efficiencies of unmodified and acetylated ATP6V0E1 mRNA were compared using a cell‐free rabbit reticulocyte lysate translation system. The results showed that NAT10‐catalyzed ac4C modification enhances the translation efficiency of ATP6V0E1 in vitro (Figure 2L). These data collectively uncovered that ATP6V0E1 is a previously uncharacterized downstream substrate of NAT10‐mediated ac4C modification.

### 2.3 High Expression of ATP6V0E1 Is Correlated with Poor Survival of Patients and Promotes Lysosomal Acidification and Metastasis

We next examined the clinical significance of ATP6V0E1 in ESCC. By performing immunohistochemical staining of ATP6V0E1 in a tissue microarray consisting of 211 ESCC tissues and 149 adjacent normal tissues, we noted that the expression level of ATP6V0E1 in tumor tissues was significantly higher than that in paired normal tissues (*P* < 0.001). A further increase of ATP6V0E1 expression in metastatic tumor tissues was observed by analyzing another microarray containing 40 paired primary tumors and matched metastatic tumor tissues (*P* < 0.05; Figure 2M). Based on Immunohistochemistry analysis, we further investigated the association between ATP6V0E1 expression and key clinicopathological parameters, including age, gender, T stage, N stage, and tumor grade. The results revealed several important associations: ATP6V0E1 expression levels were significantly elevated in patients with lymph node metastasis (N1/N2/N3) compared to N0 patients (no lymph node metastasis) (*P* < 0.05); tumors from Grade III/IV patients exhibited elevated ATP6V0E1 expression compared to Grade I/II cases (*P* < 0.05) (Table S2, Supporting Information). Moreover, Kaplan‐Meier survival analysis revealed that patients with high tumor ATP6V0E1 expression had a significantly shorter survival time (median survival time = 12 months) compared to patients with low tumor ATP6V0E1 expression (median survival time = 51 months) (log‐rank test, *P* < 0.001; Figure 2N). Consistently, further analysis using The Cancer Genome Atlas (TCGA) datasets found that ATP6V0E1 expression was significantly elevated in primary esophageal carcinoma and was positively correlated with lymph node metastasis status (Figure S4A,B, Supporting Information). Moreover, survival analysis indicated that patients with higher ATP6V0E1 expression tended to have shorter overall survival (Figure S4C, Supporting Information). Furthermore, a strong positive correlation between NAT10 and ATP6V0E1 was noted in ESCC (Pearson χ^2^ test, *P* < 0.01; Figure 2O),^[^ ^5^ ^]^ supporting our above‐mentioned finding that NAT10 positively regulates ATP6V0E1.

**Figure 3:**
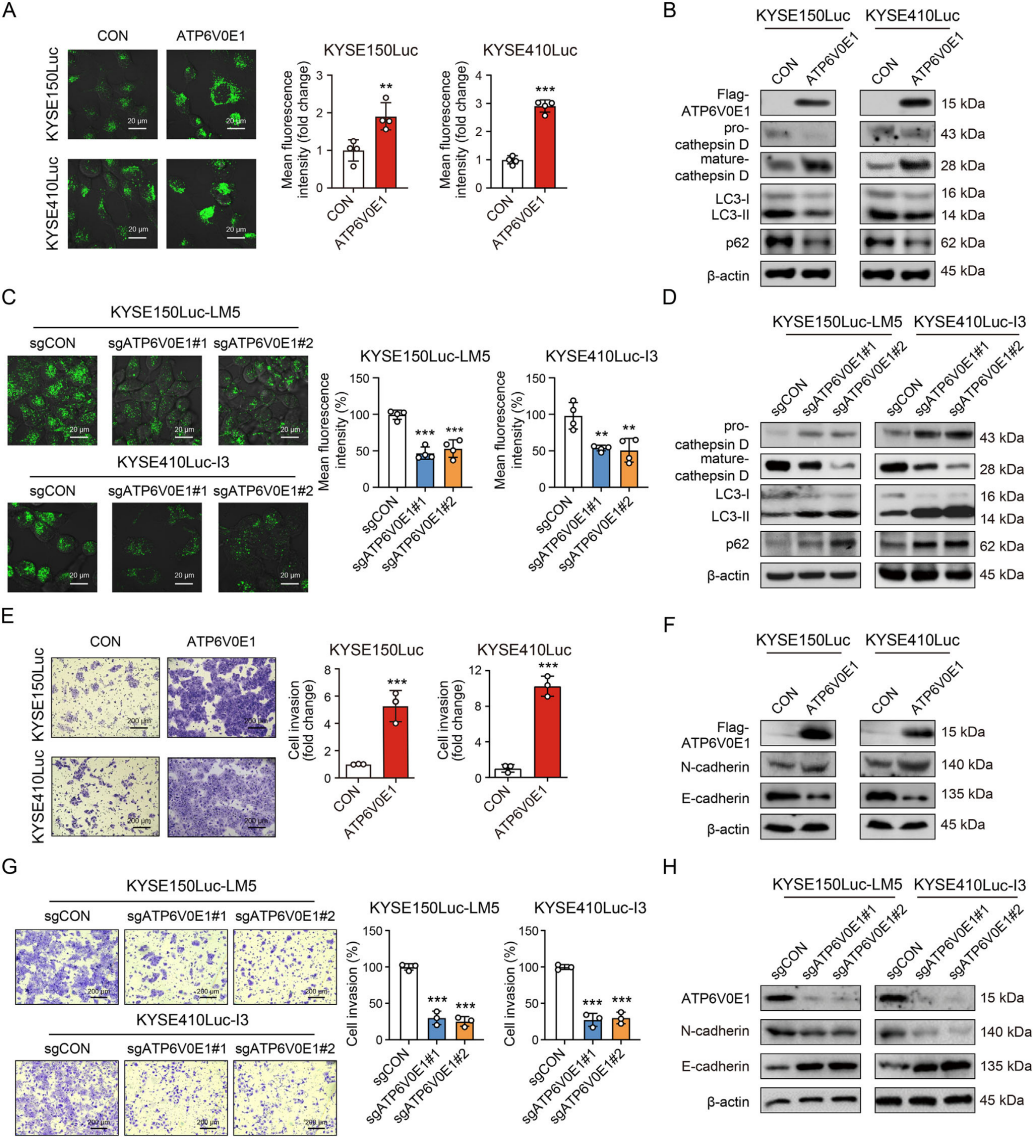
ATP6V0E1 promotes lysosomal acidification and cancer metastasis. A) LysoTracker‐Green staining was used to evaluate the lysosomal acidification status in KYSE150Luc and KYSE410Luc cells overexpressing ATP6V0E1 or vector control. Scale bar: 20 µm. B) Western blot revealing the expression levels of pro‐cathepsin D, mature‐cathepsin D, LC3, and p62 in ESCC cells overexpressing ATP6V0E1 or vector control. C) Live imaging of LysoTracker‐Green dye detecting lysosomal acidification in ESCC cells with control or ATP6V0E1 knockout. Scale bar: 20 µm. D) Western blot analysis revealing the expression levels of pro‐cathepsin D, mature‐cathepsin D, LC3, and p62 in ATP6V0E1‐knockout ESCC cells and control. E) Transwell assay comparing cell invasion between ATP6V0E1‐overexpressing and control cells. Scale bar: 200 µm. F) Western blot showing the expression of E‐cadherin and N‐cadherin in ATP6V0E1‐overexpressing or control cells. G,H) The cell invasion and EMT phenotypes were detected when ATP6V0E1 was knocked out. Scale bar: 200 µm. Bars, SDs; ** *P* < 0.01; *** *P* < 0.001.

To study the biological function of ATP6V0E1 in cancer progression, we established ESCC cell lines with ATP6V0E1 overexpression or knockout. The data from LysoTracker staining and Western blot analysis showed that overexpression of ATP6V0E1 not only increased lysosomal acidification (**Figure** **3**A), but also promoted the maturation of cathepsin D, accompanied by the reduction of LC3‐II and p62 (Figure 3B). The opposite results were obtained in ATP6V0E1‐deficient ESCC cells (Figure 3C,D). More importantly, overexpression of ATP6V0E1 led to a significant increase in cell invasion and a boost in Epithelial‐Mesenchymal Transition (EMT) phenotypes (Figure 3E,F), while a reduction was detected in ATP6V0E1‐knockout cells (Figure 3G,H). These data collectively suggested that ATP6V0E1 may serve as a novel diagnostic and prognostic biomarker for ESCC, and play an important role in promoting lysosomal acidification and cancer metastasis.

### 2.4 NAT10 Induces the Lysosomal Degradation of E‐Cadherin to Promote Cancer Metastasis via Upregulation of ATP6V0E1

**Figure 4:**
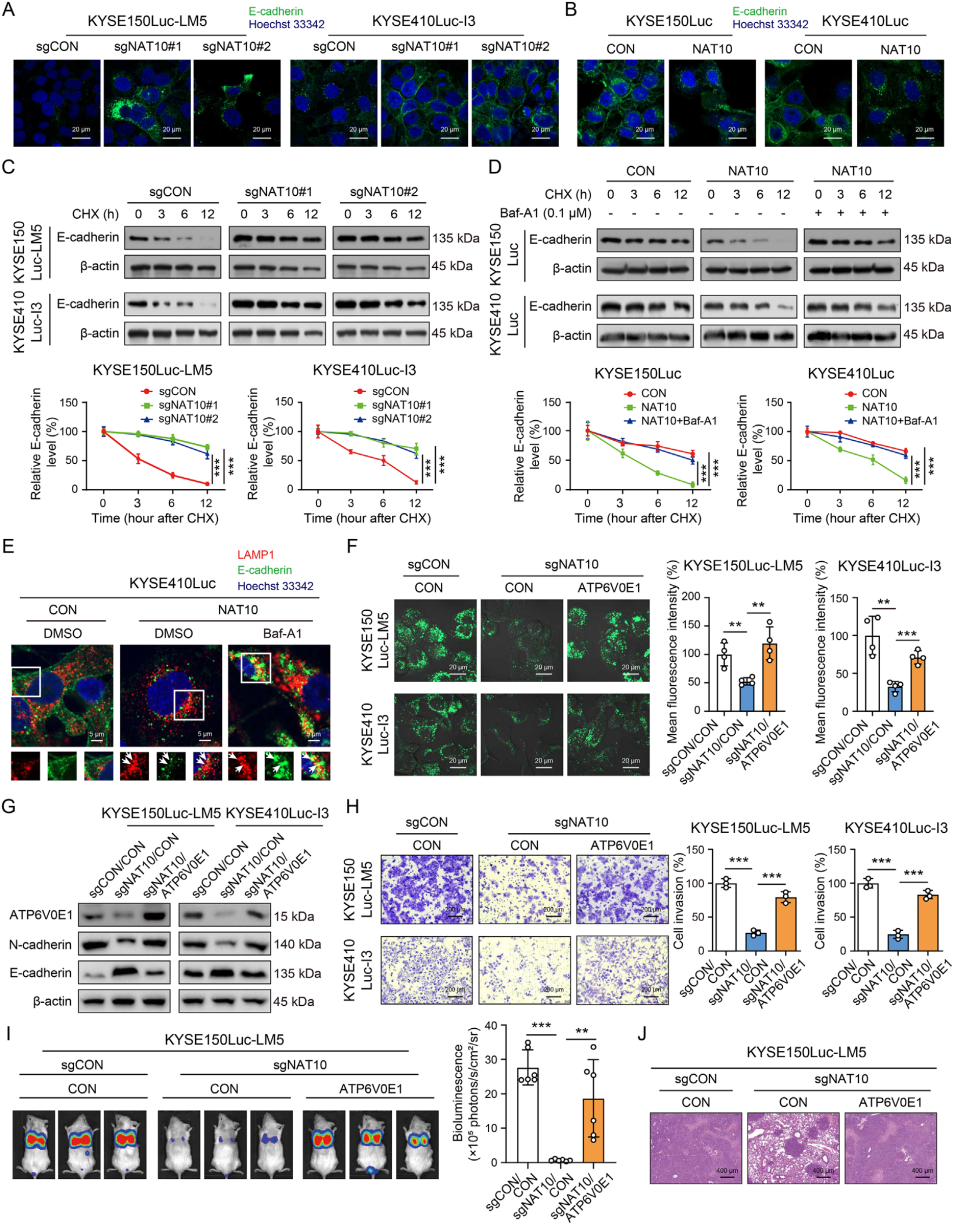
NAT10 induces the lysosomal degradation of E‐cadherin to promote cancer metastasis via upregulation of ATP6V0E1. A,B) Immunofluorescence showing the effects of NAT10 on the expression of E‐cadherin. Scale bar: 20 µm. C) Western blot detection of E‐cadherin expression in NAT10‐knockout ESCC cells pretreated with CHX (50 µg mL^−1^) for different durations (0, 3, 6, and 12 h). D) In the presence of Bafilomycin A1 (Baf‐A1, 0.1 µm) or CHX (50 µg mL^−1^), the expression of E‐cadherin is shown in NAT10‐overexpressing cells or control. E) The co‐localization of E‐cadherin with LAMP1 was detected by confocal microscopy in NAT10‐overexpressing ESCC cells or control in the absence or presence of Baf‐A1. The white arrows represent the co‐localization of E‐cadherin and LAMP1. Scale bar: 5 µm. F) LysoTracker‐Green staining shows that ATP6V0E1 mediated the effect of NAT10 on lysosomal acidification status in NAT10‐knockout KYSE150Luc‐LM5 and KYSE410Luc‐I3 cells. Scale bar: 20 µm. G,H) Western blot analysis (G) and the Boyden chamber assay (H) indicated that overexpression of ATP6V0E1 attenuated the effect of NAT10 on invasion and EMT phenotypes. Scale bar: 200 µm. I,J) Bioluminescence imaging and quantification show that ATP6V0E1 abolishes the effect of NAT10 on lung metastasis. Scale bar: 400 µm. Bars, SDs; ** *P* < 0.01; *** *P* < 0.001.

As a key component of the adherent junctions and core protein maintaining the epithelial phenotype of cells during EMT,^[^ ^17^ ^]^ the alteration of E‐cadherin protein warrants investigation. It has been shown that a pool of E‐cadherin on the cell surface undergoes constitutive internalization and subsequent endocytosis into lysosomes or recycling to maintain its presence at the plasma membrane.^[^ ^18^ ^]^ Results from immunofluorescence staining demonstrated that knockout of NAT10 led to increased expression of E‐cadherin, mainly located in the cytoplasm and on the plasma membrane, compared with the control group (**Figure** **4**A). However, most E‐cadherin lost its membrane location and only a small amount of detectable E‐cadherin was found in the cytoplasm of NAT10‐overexpressed cells compared to the control group (Figure 4B). The fluorescence intensity of E‐cadherin proteins on the membrane was analyzed and presented in Figure S5A,B (Supporting Information). More importantly, in the presence of cycloheximide (CHX), a protein synthesis inhibitor, the protein stability of E‐cadherin was found to be improved in NAT10‐deficient cells, suggesting that NAT10 regulates the expression level of E‐cadherin by altering its protein stability (Figure 4C). Next, the mechanism by which NAT10 causes E‐cadherin downregulation was further explored. We hypothesized that E‐cadherin could be degraded by lysosomes, and the results showed that treatment with Bafilomycin A1, a well‐known lysosomal acidification inhibitor that works by inhibiting v‐ATPase, can prevent the destabilization of E‐cadherin in NAT10‐overexpressing cells, thereby inhibiting the rapid downregulation of its expression (Figure 4D). Furthermore, immunofluorescence was performed using LAMP1 as a marker for the lysosome. In control cells, E‐cadherin is primarily localized to the cell membrane (left panel, Figure 4E; Figure S5C, Supporting Information). It can be observed that the distribution of E‐cadherin in the plasma membrane of the NAT10‐overexpressing cells was limited, with co‐localization observed with lysosomes. In addition, most of the E‐cadherin was degraded, resulting in weak green fluorescence (middle panel, Figure 4E; Figure S5C, Supporting Information). However, treatment of NAT10‐overexpressing cells with Bafilomycin A1 increased the amount of E‐cadherin, resulting in stronger green fluorescence that co‐localized with the lysosome marker LAMP1 (right panel, Figure 4E; Figure S5C, Supporting Information). These findings suggested that NAT10 overexpression leads to a decrease in E‐cadherin through lysosomal degradation, which was reduced in cells with NAT10 deficiency due to the inhibition of lysosomal acidification.

We next investigated whether ATP6V0E1 mediates the effect of NAT10 on cancer metastasis. First, overexpression of ATP6V0E1 alleviated the upregulated expression of E‐cadherin induced by NAT10 knockout (Figure S5D, Supporting Information), while depletion of ATP6V0E1 reinstated the reduced expression of E‐cadherin caused by NAT10 overexpression (Figure S5E, Supporting Information). Moreover, overexpression of ATP6V0E1 also counteracted the inhibitory effect of NAT10 knockout on lysosomal acidification, EMT‐associated proteins, and invasion of ESCC cells (Figure 4F–H). Conversely, ATP6V0E1 deficiency abolished the promoting effect of NAT10 on lysosomal acidification, EMT‐associated proteins, and invasion of ESCC cells (Figure S5F–H, Supporting Information). By establishing an experimental metastasis mouse model through intravenous injection of ESCC cells into NOD‐Prkdc^em26Cd52^ Il2rg^em26Cd22^ (NCG) mice, we observed that NAT10 depletion resulted in a successful delay of lung metastasis indicated by weaker bioluminescence signals, whereas overexpression of ATP6V0E1 recovered the metastatic ability of ESCC cells (Figure 4I,J). The results from the Western blot and immunohistochemical staining assays demonstrated an upregulation of E‐cadherin expression and a downregulation of ATP6V0E1 expression in the lung metastatic tumor tissues of the NAT10 knockout group compared to the control group, while overexpression of ATP6V0E1 diminished the regulatory effect of NAT10 knockout on both E‐cadherin and ATP6V0E1 expression (Figure S5I,J, Supporting Information). To further investigate whether ATP6V0E1 mediated the protein degradation in cancer cells, we performed a Western blot assay, which showed that ATP6V0E1 did not affect the total level of ubiquitin protein (Figure S6A, Supporting Information). Next, we conducted specific enrichment of ubiquitination peptides prior to high‐resolution liquid chromatography‐tandem mass spectrometry (LC‐MS/MS) analysis to map the landscape of protein degradation in ATP6V0E1‐overexpressing cells. This analysis quantified 5172 ubiquitination sites (Table S3, Supporting Information). Based on significant changes in abundance, we identified 91 upregulated sites (Fold Change > 1.5 and *P* value < 0.05) and 108 downregulated sites (Fold Change < 0.667 and *P* value < 0.05) following ATP6V0E1 overexpression (Figure S6B, Supporting Information). Notably, there were no significant differences in the modification levels of ubiquitination sites on E‐cadherin (Table S3, Supporting Information). Furthermore, the results from Figure S6C (Supporting Information) showed that the degradation of E‐cadherin increased over time with the CHX addition in ATP6V0E1 overexpression conditions (middle), while E‐cadherin degradation was not rescued in the presence of MG132, a proteasome inhibitor (right). These data demonstrated that ATP6V0E1‐mediated lysosomal acidification, rather than the proteasome pathway, is essential for the degradation of E‐cadherin. Collectively, we provided evidence that NAT10 promotes lysosomal degradation of E‐cadherin and cancer metastasis in an ATP6V0E1‐dependent manner.

### 2.5 Identification of G‐749 as a NAT10 Inhibitor to Enhance Ubiquitin‐Dependent Degradation of NAT10 via Interaction with USP39

**Figure 5:**
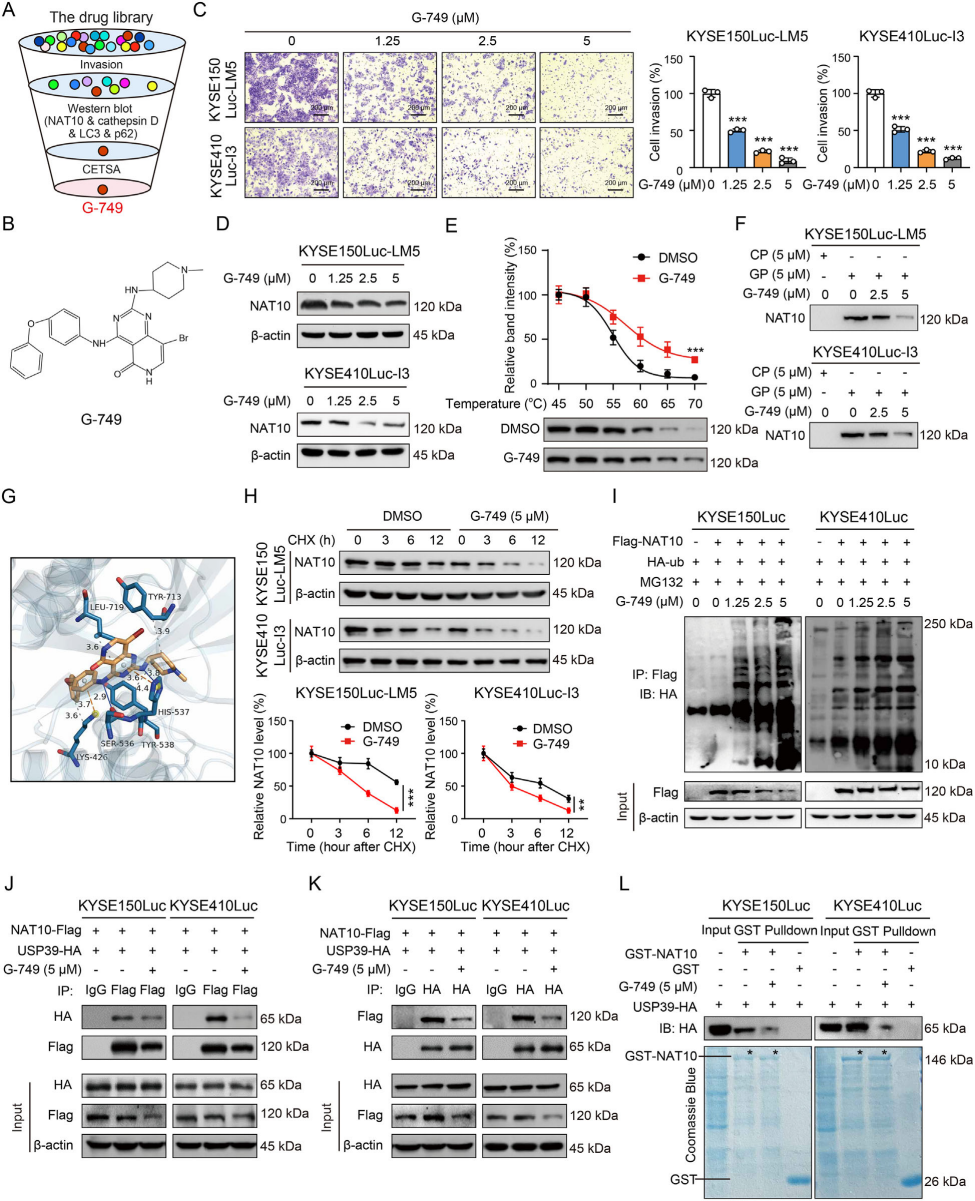
Identification of G‐749 as a NAT10 inhibitor to enhance ubiquitin‐dependent degradation of NAT10 via interaction with USP39. A) Diagram illustrating the strategy to screen drug candidates. B) Schematic diagram of the molecular structure of G‐749. C) Invasion assay was conducted on KYSE150Luc‐LM5 and KYSE410Luc‐I3 cells after treatment with increasing doses of G‐749. Scale bar: 200 µm. D) Western blot analysis detected the expression of NAT10 protein in ESCC cells upon G‐749 treatment. E) CETSA analysis revealed the binding between the NAT10 protein and G‐749. F) NAT10 were labeled with biotin and pulled down by avidin on magnetic beads after GP treatment. A biotin‐avidin experiment was conducted. G) Molecular docking indicating the specific binding sites between G‐749 and NAT10. H) Immunoblot showing NAT10 expression in KYSE150Luc‐LM5 or KYSE410‐I3 cells pretreated with CHX (50 µg mL^−1^) for different durations (0, 3, 6, and 12 h), with or without G‐749 (5 µm) treatment. I) Immunoprecipitation analysis of NAT10‐binding ubiquitin proteins in KYSE150Luc and KYSE410Luc cells following treatment with MG132 (8 µm) or G‐749 (0, 1.25, 2.5, and 5 µm) for 24 h. J,K) KYSE150Luc and KYSE410Luc cells were co‐transfected with NAT10‐Flag and USP39‐HA plasmids, then subjected to G‐749 (5 µm) treatment. The content changes of USP39‐HA or NAT10‐Flag in immunoprecipitates were analyzed. With normal IgG as a negative control. L) GST‐NAT10 pulldown assays were performed in KYSE150Luc or KYSE410Luc cells after G‐749 incubation in vitro. Bars, SDs; ** *P* < 0.01; *** *P* < 0.001.

To identify compounds that target NAT10 to inhibit cancer metastasis, a compound library consisting of 91 autophagy‐associated small molecules (Table S4, Supporting Information) was experimentally screened using transwell assay, Western blot and cellular thermal shift assay (CETSA) (**Figure** **5**A). G‐749 became our focus of this study (Figure 5B), because it satisfied all the following criteria: 1) it exhibited a significant anti‐invasive effect on ESCC cells in a dose‐dependent manner (Figure 5C; Figure S76A, Supporting Information); 2) it demonstrated a remarkable effect on the markers of autophagy and lysosomal acidification compared to other candidate compounds (Figure S7B,C, Supporting Information); 3) it could suppress the protein expression of NAT10 in a dose‐dependent manner (Figure 5D); 4) it protected the protein degradation of NAT10 (Figure 5E). Moreover, a biotin‐avidin experiment^[^ ^19^ ^]^ was performed, in which NAT10 were biotinylated with biotin and pulled down by avidin on streptavidin magnetic beads after G‐749 probe (GP) treatment. Competition experiments showed that free G‐749 inhibited the probe's ability to pull down NAT10 in a dose‐dependent manner (Figure 5F). Our results revealed that G‐749 might directly interact with NAT10. As suggested by molecular docking, the binding sites of G‐749 and NAT10 involve hydrophobic interactions at Lys‐426, Tyr‐538, Tyr‐713, and Leu‐719, π‐positive forces at Lys‐426 and His‐537, and form hydrogen bonds with Ser‐536 and His‐537 (Figure 5G). Based on the results from molecular docking, we further constructed mutant plasmids at six binding sites of G‐749 on NAT10 by mutating specific residues into alanine through point mutation experiments. The NAT10‐deficient cells were overexpressed with six different mutants NAT10 and subsequently treated with G‐749 (Figure S7D, Supporting Information). The results from a thermal shift assay revealed that mutations at the Ser‐536 and His‐537 sites eliminated the binding of NAT10 to G‐749, resulting in no significant difference in the stability of NAT10 following treatment with G‐749 compared to DMSO treatment. These data collectively suggested that the Ser‐536 and His‐537 sites are essential for G‐749 targeting NAT10.

We next investigated how G‐749 decreases NAT10 expression, while the mRNA level of NAT10 didn't change upon G‐749 treatment (Figure S7E, Supporting Information). As illustrated in Figure 5H, the results from the CHX assay showed that G‐749 decreased the protein half‐life of NAT10 in ESCC cells. Next, we sought to clarify the mechanism by which G‐749 affects the protein stability of NAT10. First, we investigated whether G‐749 regulates NAT10 protein stability through the proteasome or lysosomal pathway. As shown in Figure S8A,B (Supporting Information), G‐749 treatment enhanced the destabilization of NAT10, the rescue effect was observed in the presence of MG132, but not chloroquine. Moreover, in the presence of the proteasome inhibitor MG132, we conducted a comparison of the ubiquitination level of NAT10 in ESCC cells treated with or without G‐749 using immunoprecipitation. We noted a significant enhancement in the ubiquitination level of NAT10 after treatment with G‐749, compared to the control group (Figure 5I), supporting our hypothesis that G‐749 promotes the ubiquitin‐mediated degradation of NAT10. These results suggested that the proteasome pathway contributes to the degradation of NAT10 caused by G‐749.

Our previous studies have shown that the deubiquitin ligase Ubiquitin‐specific Peptidase 39 (USP39) directly interacts with NAT10 in a molecule‐specific manner to enhance the stability of NAT10.^[^ ^5^ ^]^ The results from co‐immunoprecipitation demonstrated a strong interaction between exogenous expression of NAT10 and USP39, whereas the interaction was weakened by G‐749 (Figure 5J,K). The same results were obtained through co‐immunoprecipitation experiments with endogenous NAT10 or USP39 protein (Figure S8C,D, Supporting Information). Moreover, the GST pulldown assay showed that G‐749 treatment reduces the in vitro binding of USP39 to NAT10 protein (Figure 5L; Figure S8E, Supporting Information). Taken together, these findings indicated that G‐749 disrupts the binding between NAT10 and USP39, leading to an increase in ubiquitination‐dependent degradation of NAT10.

### 2.6 G‐749 Decreases ATP6V0E1 ac4C Modification and Inhibits Lysosomal Acidification

**Figure 6:**
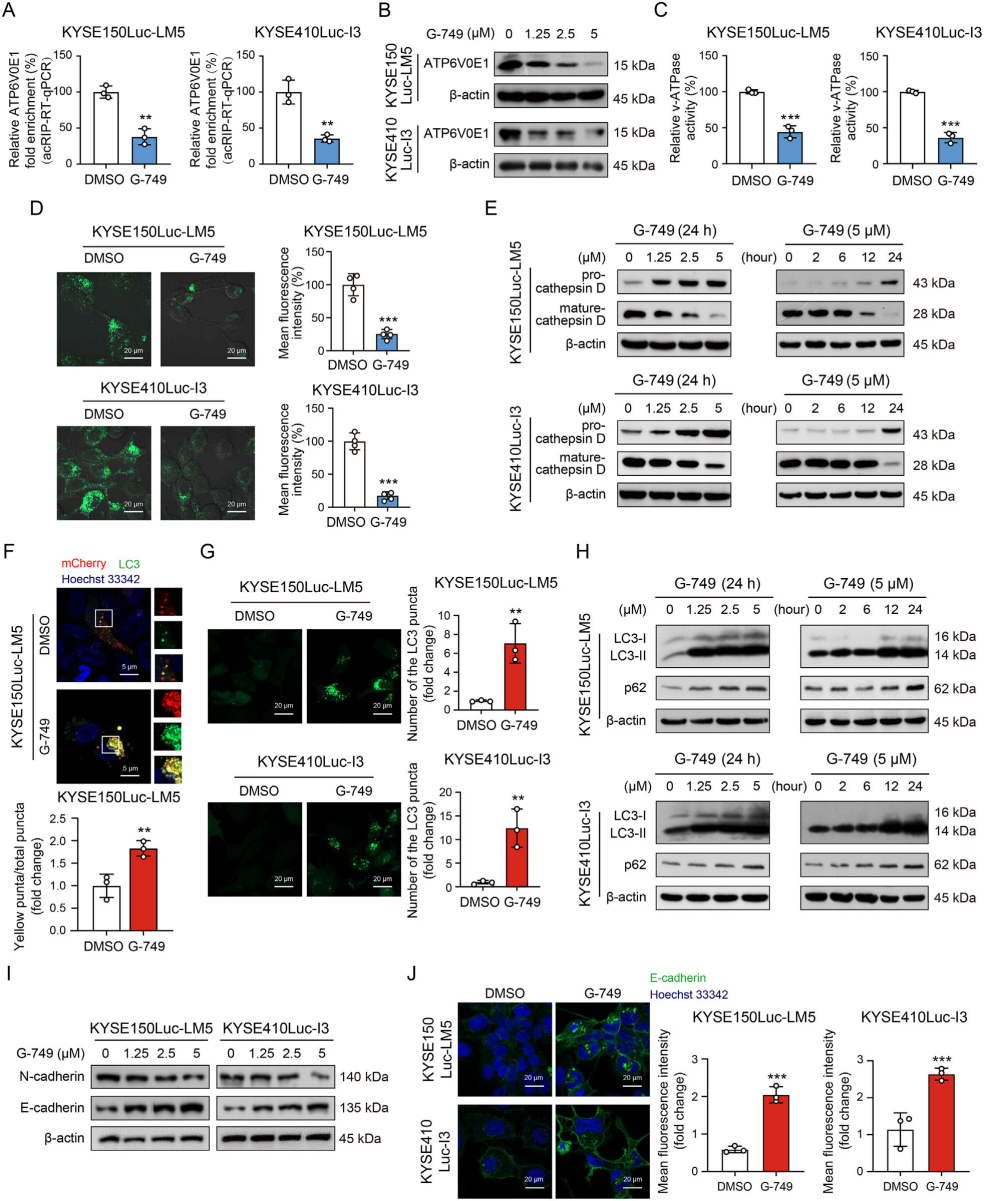
G‐749 decreases ATP6V0E1 ac4C modification and inhibits lysosomal acidification. A) acRIP‐RT‐qPCR showing the effect of G‐749 on ac4C modification of ATP6V0E1 mRNA. B) Western blot analysis of ATP6V0E1 protein in ESCC cells upon G‐749 treatment. C) V‐ATPase detection showing the effect of G‐749 on the activity of v‐ATPase in ESCC cells. D) LysoTracker‐Green staining evaluating the effects of G‐749 on lysosomal acidification in KYSE150Luc‐LM5 and KYSE410Luc‐I3 cells. Scale bar: 20 µm. E) Western blot revealing that G‐749 resulted in a decrease of the maturation of cathepsin D and an increase of its precursor form in ESCC cells in a dose‐ and time‐dependent manner. F) ESCC cells were transfected with the mCherry‐GFP‐LC3 plasmid and then treated with G‐749 for 24 h. The autophagosome (yellow) or co‐localization with lysosomes forming autolysosomes (red/yellow) was observed using confocal laser microscopy. Scale bar: 5 µm. G) ESCC cells were transfected with the GFP‐LC3 plasmid and then subjected to G‐749 intervention for 24 h. The numbers of green puncta, which represent autophagosomes, were observed using confocal microscopy. Scale bar: 20 µm. H) Western blot analysis of the protein expression of LC3 and p62 in KYSE150Luc‐LM5 and KYSE410Luc‐I3 cells after treatment with G‐749 at the indicated concentrations and time. I) Western blotting detection of E‐cadherin and N‐cadherin expression in KYSE150Luc‐LM5 cells treated with G‐749 at different concentrations. J) Immunofluorescence analysis revealing the expression of E‐cadherin upon G‐749. Scale bar: 20 µm. Bars, SDs; ** *P* < 0.01; *** *P* < 0.001.

Given the important role of NAT10 in ac4C modification and lysosomal dysregulation, we further examined the ATP6V0E1 ac4C modification and lysosomal function after treatment with G‐749 targeting NAT10. The acRIP‐RT‐qPCR analysis revealed that G‐749 treatment markedly reduced the ac4C modification level of ATP6V0E1 mRNA in ESCC cells (**Figure** **6**A). As shown in Figure 6B, the protein levels of ATP6V0E1 decreased in a dose‐dependent manner as a consequence of G‐749 intervention. Next, we found that G‐749 leads to a significant reduction in v‐ATPase activity (Figure 6C). Furthermore, a LysoTracker staining assay was performed, showing that exposure to G‐749 caused a notable decrease in lysosomal acidification (Figure 6D). The aforementioned results, combined with the experimental findings in which G‐749 treatment led to a significant reduction in mature‐cathepsin D (Figure 6E), shed light on the biological function of G‐749 in lysosomal function. We further employed mCherry‐GFP‐LC3 double fluorescence tandem plasmids (mCherry: red; GFP: green). The green fluorescence signal emitted by GFP was quenched at a lower pH value. In contrast, mCherry (red) could emit red fluorescence and was less affected by changes in pH. As shown in Figure 6F, the co‐localization of red and green signals resulted in prominent yellow highlights in esophageal cancer cells following G‐749 treatment. The green fluorescence intensity of GFP‐LC3‐labeled autophagosomes significantly increased after G‐749 treatment (Figure 6G). Western blot analysis also revealed that G‐749 induced an accumulation of LC3‐II and p62 in a dose‐ and time‐dependent manner (Figure 6H), supporting the idea that G‐749 may interfere with the acidity of lysosomes to increase the number of autophagosomes. Additionally, we observed a significant increase in the expression of E‐cadherin and a decrease in the levels of N‐cadherin in ESCC cells following the G‐749 intervention (Figure 6I). We also studied the localization of E‐cadherin after treatment with G‐749. Immunofluorescence analysis revealed that treatment with G‐749 resulted in an increased expression of E‐cadherin, mainly localized on the cell membrane (Figure 6J). Taken together, these results indicated that G‐749 may impede the degradation function of autolysosomes by inhibiting ATP6V0E1 ac4C modification during late autophagic flux, leading to an increase in the expression of E‐cadherin.

### 2.7 NAT10 Is Required for the Bioactivity of G‐749 in Suppressing Lysosomal Dysregulation and Tumor Metastasis

**Figure 7:**
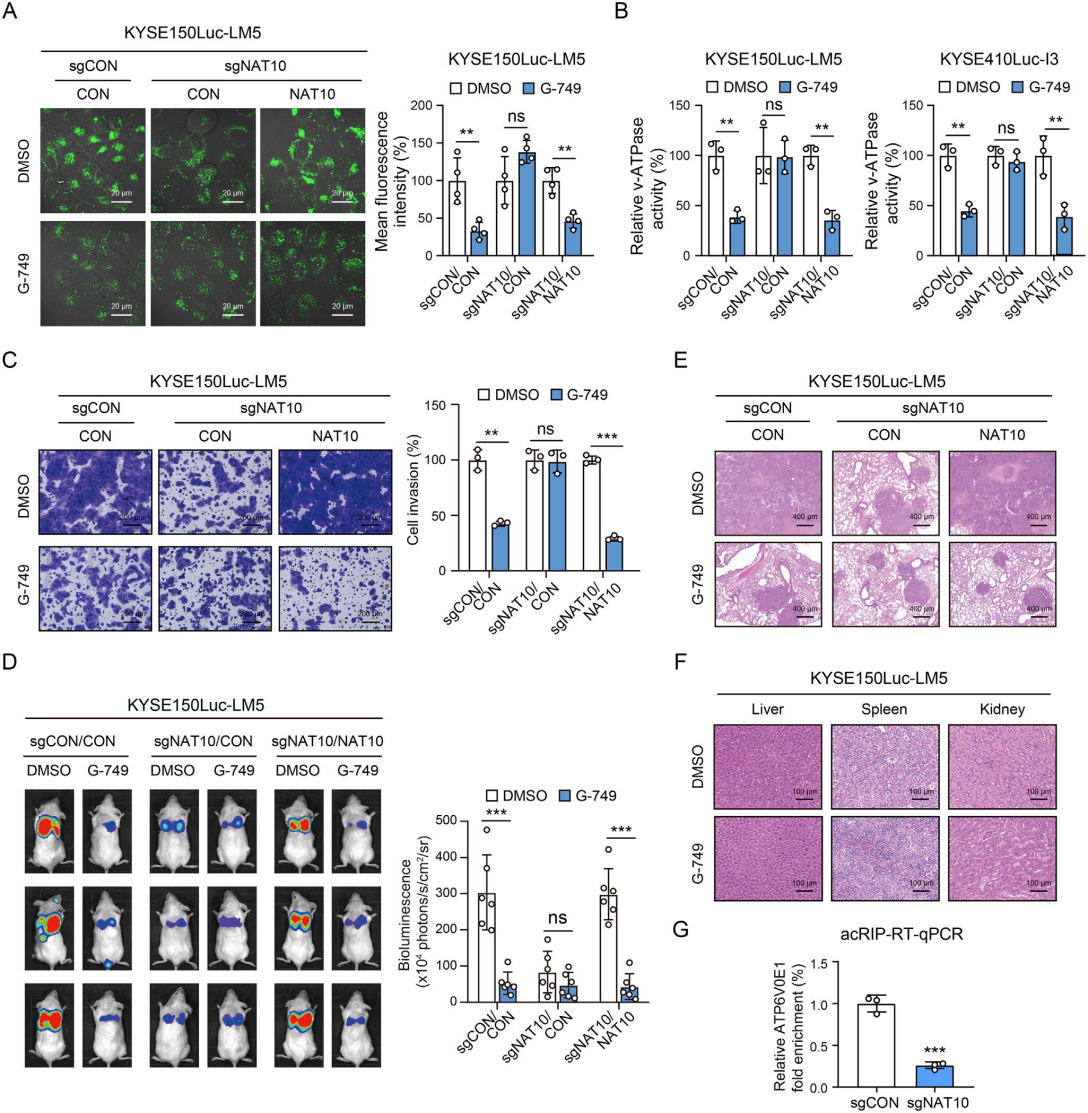
NAT10 is required for the bioactivity of G‐749 in suppressing lysosomal dysregulation and tumor metastasis. A) LysoTracker‐Green staining showing the inhibitory effect of G‐749 on lysosomal acidification, while not in NAT10‐knockout group, and that the suppressive effect of G‐749 was restored when the cells were reconstituted with NAT10. Scale bar: 20 µm. B) Detection of v‐ATPase activity in ESCC cells as indicated. C) Invasion chamber assay comparing the invasive ability of NAT10‐knockout ESCC cells that were further transfected with NAT10 or control in the presence or absence of G‐749. Scale bar: 200 µm. D) Bioluminescence imaging and quantification of lung metastasis showing the inhibitory effect of G‐749 on tumor metastasis as indicated. E) Hematoxylin and eosin (H&E) staining of lung sections as indicated. Scale bar: 400 µm. F) Histological analysis of major organs in the groups. G) The ac4C modification level of ATP6V0E1 mRNA in lung metastatic tumor tissues was assessed by acRIP‐RT‐qPCR. Scale bar: 100 µm. Bars, SDs; ns, no significance; ** *P* < 0.01; *** *P* < 0.001.

To investigate the importance of NAT10 in the biological activity of G‐749, NAT10‐deficient ESCC cell lines were established. Our data indicated that G‐749 can reduce lysosomal acidity and v‐ATPase activity in ESCC cells, but this phenotype could not be observed when NAT10 was knocked out. When we re‐overexpressed wild‐type NAT10 into the NAT10‐knockout cells, the effect of G‐749 on the lysosomal acidity and v‐ATPase activity was recovered (**Figure** **7**A,B), suggesting the essential role of NAT10 in the bioactivity of G‐749. We next investigated the role of NAT10 in the inhibitory effect of G‐749 on invasion and metastasis. The Boyden chamber invasion assay revealed that G‐749 did not significantly inhibit the invasive ability of NAT10‐knockout ESCC cells. The decreased sensitivity of NAT10‐knockout ESCC cells to G‐749 was remarkably restored in the cells re‐overexpressing NAT10 (Figure 7C). As illustrated in Figure 7D, administration of G‐749 (5 mg kg^−1^) resulted in a significant decrease in lung metastasis of ESCC cells. Consistent with in vitro studies, our in vivo evaluations suggested that G‐749 did not exert a remarkable effect on the metastasis of NAT10‐deficient cells in mice. However, the inhibitory effects of G‐749 on lung metastasis were restored in mice when the NAT10‐deficient ESCC cells were further re‐overexpressed with NAT10 (Figure 7D,E). The morphology of major organs indicated that G‐749 did not exhibit organ toxicity in vivo (Figure 7F). No significant differences in the levels of alanine aminotransferase (ALT), aspartate aminotransferase (AST), and other blood indices were observed in G‐749‐treated mice (Figure S9, Supporting Information). Moreover, ac4C modification of ATP6V0E1 was also observed in lung metastatic tumor tissues from mice, while significantly decreased in the NAT10‐deficient group (Figure 7G). These data collectively demonstrated that G‐749 could be a novel therapeutic strategy against cancer by targeting NAT10.

## 3 Discussion

**Figure 8:**
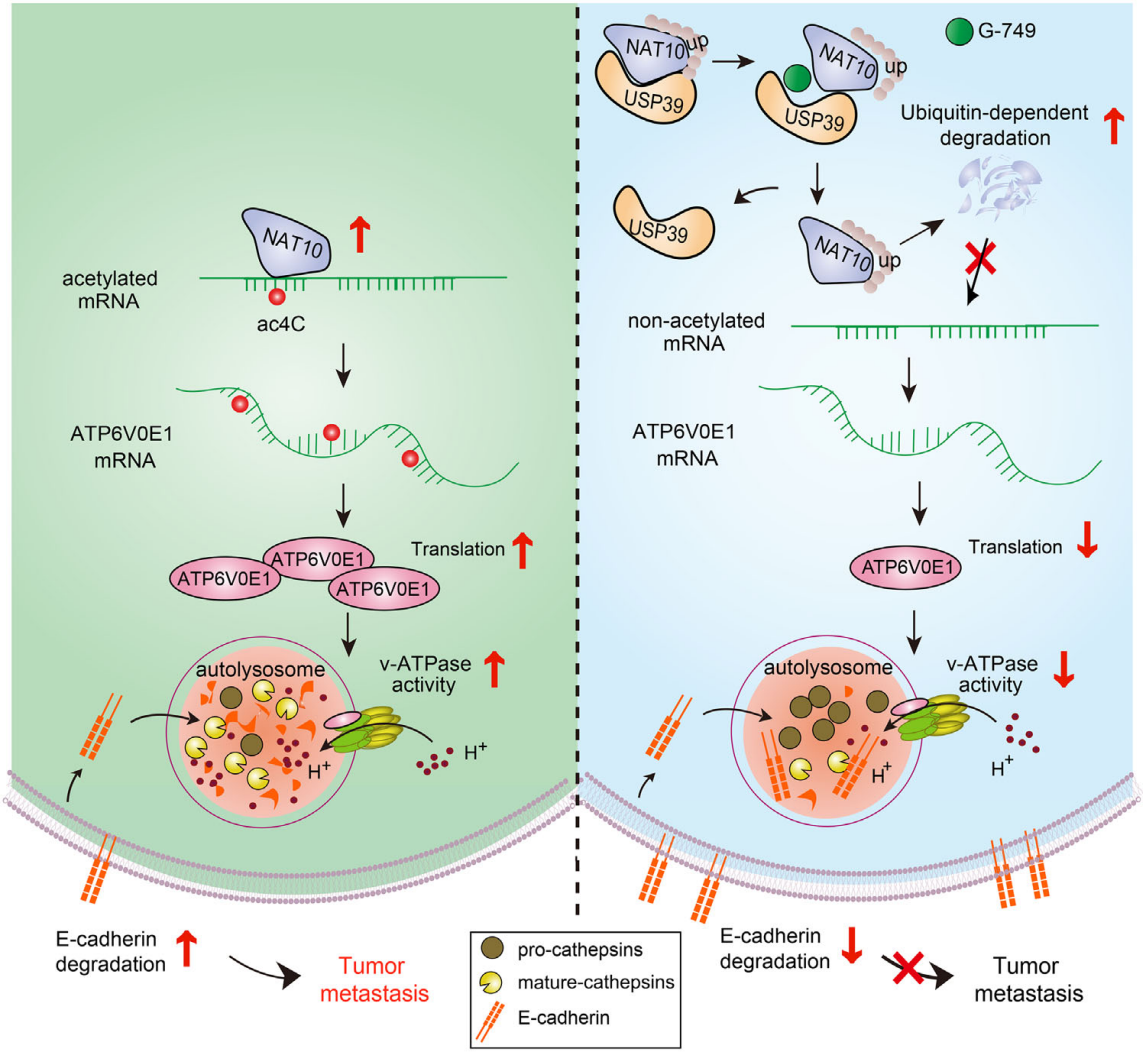
Schematic model.

Here, we provide the first evidence showing the biological function of NAT10‐catalyzed ac4C modification in regulating autophagy‐associated lysosomal acidification. Moreover, ATP6V0E1 was identified as a crucial substrate for ac4C modification, and may be a prognostic biomarker and functional target for tumor metastasis. The discovery and characterization of G‐749 as a NAT10 inhibitor to suppress lysosomal dysregulation and tumor metastasis opens up new avenues for cancer drug development (**Figure** **8**).

First, we demonstrated the critical role of ATP6V0E1 in tumor metastasis, in particular, the malignant development of ESCC, for the first time. ESCC is one of the most aggressive malignancies worldwide, with an overall 5‐year survival rate of less than 20%. Metastasis is the major cause of cancer mortality, and accounts for ≈90% of cancer deaths.^[^ ^20^ ^]^ However, the molecular mechanism underlying metastasis caused by lysosomal acidification remains largely unknown. V‐ATPase, a multimeric complex comprising the cytoplasmic V1 domain and the V0 domain, has garnered sustained attention for its important role in regulating lysosomal function.^[^ ^11^, ^21^ ^]^ Recently, several v‐ATPase subunits, such as ATP6L and ATP6V1A, have been suggested to alter the lysosomal function and participate in tumorigenesis.^[^ ^22^ ^]^ However, as an important v‐ATPase assembly subunit, the biological function of ATP6V0E1 in tumor metastasis has not been explored. Our in vitro and in vivo results showed that overexpression of ATP6V0E1 can dramatically promote esophageal cancer invasion and metastasis through regulation of E‐cadherin. Notably, as the gatekeeper and marked feature of EMT process, the regulatory mechanisms underlying the downregulation of epithelial marker E‐cadherin protein warrant investigation. Here, we found that ATP6V0E1 accelerates the lysosomal degradation of E‐cadherin, and ultimately triggers the ESCC invasion and metastasis. Tumor metastasis, however, is a multifaceted process involving numerous factors and steps. Beyond EMT and E‐cadherin expression investigated in this study, a multitude of factors influence tumor metastasis, including the tumor microenvironment, lymphangiogenesis, and angiogenesis. The potential interplay among these crucial factors warrants further systematic investigation. Furthermore, the clinical relevance analysis revealed that ATP6V0E1 was upregulated in ESCC tissues and further increased in metastatic tumor tissues. More importantly, high ATP6V0E1 expression was found to be associated with poor patient survival. In combination with the above can be seen, targeting NAT10 or ATP6V0E1 for the treatment of esophageal cancer patients presents certain feasibility.^[^ ^5^, ^23^ ^]^ Future research must delve deeper into the mechanistic roles of these two targets in esophageal cancer, develop more specific and effective inhibitors, and explore combinatorial treatment strategies to enhance therapeutic outcomes for esophageal cancer. Furthermore, the output of this study may expand the opportunities to study lysosomes and cancer progression, and thus provide a theoretical basis for further individualized diagnosis and treatment strategies for cancer.

Second, the biological function of NAT10‐catalyzed ac4C modification in regulating lysosomal function was uncovered for the first time. Emerging studies have shown that epitranscriptomic RNA modifications play a crucial role in processes such as cell differentiation, embryonic development, drug resistance, and tumorigenesis through post‐transcriptional gene regulation.^[^ ^3^, ^24^ ^]^ For instance, YBX1 facilitates the ESCC progression via m5C‐dependent SMOX mRNA.^[^ ^25^ ^]^ However, as a newly identified epigenetic modification within mRNA, the biological function of ac4C modification remains largely unknown. Interestingly, NAT10 has been identified as the only known writer enzyme for ac4C modification on mRNAs. Notably, the function of NAT10‐catalyzed ac4C modification in regulating autophagy and lysosomal acidification has never been reported. According to our findings from gain‐ and loss‐of‐function experiments, NAT10 induced lysosomal acidification by regulating the translation efficiency of ATP6V0E1, which, in turn, affected the autophagy process. A series of in vitro and in vivo functional studies further confirmed that ATP6V0E1 mediates the effect of NAT10 on lysosomal degradation of E‐cadherin, cell invasion, and tumor metastasis. The above data not only enriched our understanding of ac4C modification in lysosomal biogenesis, but also proposed a novel druggable mechanism via inhibition of NAT10. In general, the current research on ac4C modification, lysosomal acidification, and metastasis of esophageal cancer is still in its preliminary stage. However, these studies offer a novel avenue for comprehending the underlying mechanisms of esophageal cancer metastasis and present potential targets and strategies for therapeutic intervention.

Third, we identified G‐749 as a potential inhibitor to suppress cancer metastasis by impairing lysosomal acidification. Drugs targeting RNA modification were proposed as a novel strategy for tumor therapy.^[^ ^26^ ^]^ For example, the FTO inhibitor R‐2‐hydroxyglutarate exerts anti‐tumor effects by inhibiting the proliferation of tumor cells with high expression of FTO through the FTO/m6A/MYC/CEBPA signaling pathway.^[^ ^27^ ^]^ However, there are still few targeted drugs for NAT10‐mediated ac4C modification. In this study, G‐749 was identified as a novel compound that could impair lysosomal acidification and inhibit esophageal cancer metastasis. Further pharmacological research indicated that G‐749 significantly weakened the interaction between NAT10 and the deubiquitinating ligase USP39, leading to the degradation of NAT10 in a proteasome‐dependent manner. Our study provided direct evidence that G‐749 could affect the mRNA ac4C modification, protein level and activity of v‐ATPase of ATP6V0E1. It is important to note that NAT10 possesses numerous substrates. The possibility that G‐749 may also work on other substrates, in addition to ATP6V0E1, could not been excluded, which warrants our further investigation. Based on this candidate, optimization of the drug structure is expected to generate more potent and selective compounds for drug discovery.^[^ ^28^ ^]^ Collectively, we provide a new strategy for combating the metastasis of ESCC by inhibiting NAT10‐catalyzed ac4C modification and repairing the subsequent autophagy‐lysosomal dysfunction, shedding light on the treatment of this lethal disease.

## 4 Experimental Section

### Drugs and Antibodies

G‐749 (HY‐12333) and Bafilomycin A1 (Baf‐A1, HY‐100558) were bought from MCE (Chengdu, China). Cycloheximide (CHX, S7418), and MG132 (S2619) were from Selleck (Houston, TX, USA). The primary antibodies against β‐actin (3700), NAT10 (66548), E‐cadherin (14472), N‐cadherin (13116), Cathepsin D (2284), USP39 (23303), LAMP1 (9091), peroxidase‐labeled antibody to rabbit IgG (7074), peroxidase‐labeled antibody to mouse IgG (7076), anti‐mouse IgG (H + L) F(ab“)_2_ Fragment (Alexa Fluor 488 Conjugate) (4408), and anti‐rabbit IgG (H+L) F(ab”)_2_ Fragment (Alexa Fluor 594 Conjugate) (8889) were all from Cell Signaling Technology (Boston, MA, USA). Anti‐HA (sc‐53516) and Flag (sc‐166384) were obtained from Santa Cruz Biotechnology (Dallas, TX, USA). Anti‐ATP6V0E1 (ABIN7094686) was purchased from Antibodies‐Online (Aachen, Germany). Anti‐LC3 (14600‐1‐AP), p62 (66184‐1‐Ig), and N4‐acetylcytidine (ac4C, 68498‐1‐Ig) were purchased from Proteintech (Chicago, IL, USA). BiotinPEG3‐Azide (B122225) was obtained from Aladdin (Shanghai, China). TCEP (T885109) was bought from Macklin (Shanghai, China). TBTA (T7086) was purchased from TargetMol (Shanghai, China).

### Cell Culture

The human ESCC cell lines KYSE150 (DSMZ, ACC 375) and KYSE410 (DSMZ, ACC 381) were purchased from German Center for the Collection of Microbial Species (Braunschweig, Germany) and maintained with RPMI‐1640 medium (Thermo Fisher Scientific, Waltham, MA, USA) containing 10% fetal bovine serum (FBS, Invitrogen, Gaithersburg, MD, USA) at 37 °C in a 5% CO_2_ environment. The highly metastatic cell sublines KYSE150Luc‐LM5 and KYSE410Luc‐I3, derived from the luciferase (Luc)‐expressing esophageal squamous cell carcinoma cell lines KYSE150 and KYSE410, were constructed as previously described.^[^ ^5^ ^]^ All cell lines used in this study were passaged within 35 passages and validated by short tandem repeat analysis.

### Plasmids and Transfection

The mCherry‐GFP‐LC3 (110060), GFP‐LC3 (11546), and RFP‐LAMP1 (1817) plasmids were obtained from Addgene (Cambridge, MA, USA). The coding sequences of NAT10 and ATP6V0E1 were cloned into pcDNA3.1, and the NAT10 and ATP6V0E1 sgRNAs were inserted into the lentiCRISPRV2 vector (52961, Addgene). The NAT10, ATP6V0E1 expressing plasmids, and ATP6V0E1 knockout plasmid were constructed by laboratory using primers from Hanyi Biosciences Inc. (GuangZhou, China). NAT10 or ATP6V0E1 overexpressing or knockout ESCC cell lines were established using the third‐generation lentivirus packaging system (12251, 12253, and 12259, Addgene). After cells were infected with lentiviral supernatants in the presence of 5 µg mL^−1^ polybrene, screening was performed using Geneticin or puromycin (Thermo Fisher Scientific, Waltham, MA, USA).

### Tissue Microarray and Immunohistochemistry

The expression of ATP6V0E1 was detected in a tissue microarray comprising 211 cases of ESCC and 159 paired adjacent normal tissues (Shanghai Outdo Biotech, Shanghai, China), as well as in another tissue microarray containing 40 pairs of primary ESCC and matched metastatic tissues (Biomax, Rockville, MD, USA). Immunohistochemical staining was performed as described previously.^[^ ^29^ ^]^ In brief, sections were deparaffinized, rehydrated, and incubated with 3% H_2_O_2_ to block endogenous peroxidase. After being blocked with 1% BSA for 2 h, the sections were incubated with a primary antibody against ATP6V0E1 overnight at 4 °C. They were then incubated with HRP‐linked secondary antibody for 1 h and detected using 3, 3′‐diaminobenzidine (DAB) substrate (Dako, Mississauga, ON, Canada). An inverted microscope was used to observe and take images. The staining intensity scores were categorized as follows: no staining (0), weak staining (1), moderate staining (2), and strong staining (3). Samples with a score of 0 or 1 were classified as low expression of ATP6V0E1, while those with a score of 2 or 3 were classified as having high expression of ATP6V0E1.

### Immunofluorescence

The cells were spread on the glass coverslips at a density of 8×10^4^ cells per well. After being fixed with 4% paraformaldehyde, the cells were then blocked with 5% FBS containing 0.3% cell membrane permeability agent Triton X‐100 at room temperature in the dark. Then the cells were incubated with primary antibodies (anti‐LAMP1, 1:700 dilution; anti‐E‐cadherin, 1:500 dilution) overnight at 4 °C, followed by incubation with fluorescent dye‐conjugated secondary antibodies (1:500 dilution) at room temperature in dark. The nuclei were stained with Hoechst 33342 (5 µg mL^−1^, Beyotime Biotechnology) for 10 min in the dark. A laser scanning confocal microscope (LSM 800; Carl Zeiss, Jena, Germany) was used to observe and capture images.

### LysoTracker Staining

ESCC cells were inoculated onto confocal plates at a density of 1 × 10^5^ cells per well. LysoTracker Green DND‐26 (L7526; Invitrogen, Carlsbad, CA, USA) was diluted in basic RPMI‐1640 culture medium to prepare a working solution with a concentration of 50 nm. After 24 h of drug treatment, the cells were incubated in 200 µL of LysoTracker working solution at 37 °C for 20 min under dark conditions. The LysoTracker solution was then discarded, and then phosphate buffer saline (PBS) was added. Images were observed and captured using a laser confocal scanning microscope.

### V‐ATPase Activity Detection Assay

The v‐ATPase test kit (GMS50244) was purchased from Genmed Sciences (Arlington, MA, USA). The v‐ATPase activity of ESCC cells was determined following the manufacturer's instructions. Briefly, cells were inoculated in 6‐well plates, and the drug was administered 24 h later. The protein concentration was determined and adjusted to 2 µg µL^−1^. Each group of proteins was separated into two parts for the non‐specific ATPase activity assay and then mixed with the reagent provided by the kit. The absorbance was measured at 340 nm and then again after a 10‐min interval. V‐ATPase activity was calculated by subtracting non‐specific v‐ATPase activity from total v‐ATPase activity.

### In Vitro Cell Invasion Assays

The protocol for diluting matrigel (BD Biosciences) and lysing cells in an in vitro cell invasion assay was previously described.^[^ ^5a^ ^]^ In brief, 100 µL well^−1^ of the diluted matrigel was added to the upper chamber and left for 1 h at 37 °C. The drug‐containing cell suspension (4 × 10^5^ cells well^−1^) was added to the upper chamber, while cell medium containing 20% FBS was added to the bottom chamber. After 24–36 h, the cells were fixed with 4% paraformaldehyde and stained with Crystal Violet. The matrigel and cells in upper compartment were swabbed, and the number of transmembrane cells was recorded using an inverted microscope.

### Western Blot

Total protein was collected after cell lysis, quantified using BCA protein assay kits (Thermo Fisher Scientific, Waltham, MA, USA). The proteins were separated by SDS‐PAGE electrophoresis and transferred to a PVDF membrane. After blocking with 5% skim milk, the membrane was incubated with the primary antibody at 4 °C overnight, and subsequently incubated with an HRP‐conjugated secondary antibody at room temperature for 1 h. After washing with TBST, the Bio‐Rad chemiluminescence imager was used to develop the protein band images.

### RT‐qPCR

The total RNA of cells was extracted using RNA extraction kit (DP424, TianGen, Beijing, China). RNA concentration was measured using a NanoDrop microspectrophotometer. The cDNA product was obtained after reverse transcription PCR. Real‐time PCR was conducted with qPCR SuperMix (Transgen, Beijing, China) on a CFX96 touch Real‐Time PCR detection system (Bio‐Rad). All target gene expression was normalized to the GAPDH control gene. Primers are listed in Table S5 (Supporting Information). To evaluate mRNA stability, cells were treated with 5 µm actinomycin D for 0, 15, 30, 60, 90, and 120 min, and the mRNA half‐life was determined.

### acRIP‐RT‐qPCR

Total RNA was extracted as mentioned above. Acetylated RNA was enriched and subjected to immunoprecipitation according to the manufacturer's instructions (Epibiotek, Guangzhou, China). Fragmentation buffer was added to 500 µg of DNase‐treated total RNA and the reaction was stopped immediately by adding EDTA. The RNA fragments were purified and recovered using a Zymo RNA Clean and Concentrator Kit (Zymo Research, OC, USA). Anti‐ac4C antibody and cleaned protein G‐magnetic beads were mixed in a precipitation buffer and kept at 4 °C for 4 h on a rotator. After magnetic separation, the supernatant was removed and the precipitation was washed with wash buffer twice. Then 5× precipitation buffer and RNase inhibitor were added. The mixture was incubated overnight rotation at 4 °C, and washed with low‐salt buffer and high‐salt buffer twice. The acetylated RNA obtained through immunoprecipitation was purified using a HiPure cell miRNA Kit (Magen, Guangzhou, China). Immunoprecipitated RNA was subjected to one‐step reverse transcription PCR with Green One‐Step RT‐qPCR SuperMix (Transgen, Beijing, China) to detect ac4C‐modified ATP6V0E1 mRNA. Related primers are shown in Table S5 (Supporting Information).

### RNA Immunoprecipitation (RlP)

More than 2 × 10^7^ cells per sample were cross linked with 1% formaldehyde and stop crosslink by adding 125 mm Glycine. The cells were lysed in 150 mm KCl, 25 mm, Tris‐HCl (pH 7.4), 5 mm EDTA, 0.5% NP‐40, 0.5 mm DTT, with 100 U mL^−1^ RNAase inhibitor and 1× protease inhibitor cocktail and centrifuged for precipitation. Protein A/G agarose beads pre‐coated with 2 µg primary antibodies against NAT10 or normal mouse IgG were incubated with sufficient cell lysates at 4 °C overnight. And the beads containing immunoprecipitated RNA‐protein complex were treated with proteinase K to remove proteins. Then interested RNAs were purified by trizol methods and detected by RT‐qPCR with primers amplifying the ATP6V0E1 acPeak region.

### Targeted ac4C Sequencing

Targeted ac4C sequencing was conducted according to previous methods.^[^ ^30^ ^]^ In brief, total RNA was extracted and chemically processed before reverse‐transcribed into cDNA, and then PCR amplification and Sanger sequencing were performed (Rui Biotech, Beijing, China). The peak height of each base was determined, and the mismatch rate was calculated.

### In Vitro Translation Assay

The ATP6V0E1 coding sequence containing the 3′UTR region was cloned into pcDNA3.1 vectors containing a T7 promoter and HA tag, which were used as DNA templates for in vitro transcription. RNA was in vitro transcribed by T7 RNA polymerase using the MEGAscript Kit (AM1333, Thermo Fisher Scientific) according to the manufacturer's instructions. The transcribed RNA products were extracted from their action mixture by lithium chloride precipitation. Then, the in vitro ATP6V0E1 RNA acetylation assay was conducted in a standard 100 µL reaction mixture containing 50 mM HEPES‐KOH (pH 7.6), 150 mm KCl, 5 mm MgCl, 1 mm DTT, 1 mm ATP, 0.5 mm acetyl‐CoA, 4 µg ATP6V0E1 mRNA, and NAT10 protein were incubated at 37 °C for 2 h.^[^ ^31^ ^]^ After the reaction, RNA was extracted with phenol‐chloroform followed by isopropanol precipitation. The ac4C formation was analyzed by dot blot using an antibody against ac4C. Finally, the translation of ATP6V0E1 mRNA was performed by incubating 2 µg of either acetylated or unmodified mRNA templates with 35 µL of rabbit reticulocyte lysate (L4960, Promega) in a final volume of 50 µL. The efficiency of translation was determined by Western blot analysis.

### Luciferase Reporter Assay

To construct luciferase reporter plasmids, the wild‐type acPeak in ATP6V0E1 3′UTR and the mutant sequence, in which the ac4C‐modified C on ATP6V0E1 3′UTR was replaced by G, were cloned into the pmirGLO vector. KYSE150 and KYSE410 cells were inoculated in 12‐well plates at a concentration of 1 × 10^5^ cells per well and transfected with the luciferase reporter plasmids. After 48 h, the luciferase activity was measured using the Dual‐Luciferase Reporter Assay System (Promega, USA), according to the manufacturer's instructions. Translation efficiency is determined as the quotient of reporter protein production Firefly luciferase (F‐Luc)/Renilla luciferase (R‐Luc) divided by mRNA abundance. Primers for this assay are listed in Table S5 (Supporting Information).

### Proteomic Analysis of Ubiquitination

Cells covered in precooled PBS were quickly scraped onto the side of the dish using a clean cell scraper, and were subsequently aspirated into a centrifuge tube. The supernatant was removed by centrifugation. The cell precipitates were obtained and lysed into total proteins. Proteins were then reconstituted in IAP Buffer. Two glycine residues (K‐GG) were generated in the lysine modification site of the ubiquitinated protein, which was specifically enriched through pretreatment with anti‐K‐ε‐GG antibody beads (PTMScan Ubiquitin Remnant Motif (K‐ε‐GG) Kit, 5562, Cell Signaling Technology). The cells were incubated at 4 °C for 1.5 h, centrifuged at 2000×g for 30 s, and the supernatant was discarded. Anti‐K‐ε‐GG antibody beads were washed three times with IAP buffer. Then, 0.15% trifluoroacetic acid (TFA) was added to the washed beads and incubated at room temperature for 10 min. An additional 0.15% TFA was then added, followed by centrifugation at 2000×g for 30 s. The supernatant was desalting with C18 STAGE Tips. Ubiquitinated proteomics analysis was performed by data‐dependent acquisition‐synchronous cumulative continuous fragmentation (ddaPASEF) scanning mode on a timsTOF Pro mass spectrometer (Bruker) based on spectral properties (retention time, m/z ratio, and peak intensity). The MS raw data from each sample were combined and analyzed using the MaxQuant software for both identification and quantitation.

### Biotin‐Avidin Experiment

A small‐molecule fluorescent probe, referred to as the control probe (CP), whose structural formula is provided in Figure S10A (Supporting Information), was added to the small‐molecule monomer G‐749 to obtain the modified molecule G‐749 probe (GP), with its structural formula presented in Figure S10B (Supporting Information). The cells (2 × 10^6^ cells well^−1^) were cultured in 100 mm dishes. After incubation with CP or GP (5 µm) for 6 h, UV irradiation (wavelength: 365 nm) for 30 min. After the cells were subsequently collected, concentrations were determined using an enhanced BCA assay kit. A 200 µg sample (1 mg mL^−1^) was extracted for click reaction. Biotin‐peg3‐azide (400 µm), TBTA (100 µm), TCEP (1 mm), and CuSO_4_ (1 mm) were supplemented to the cell lysates. Incubate at room temperature for 3 h. Then, the biotin‐labeled proteins were consequently precipitated with acetone. The protein was re‐suspended by PBS, followed by the addition of 50 µL Dynabeads M‐280 Streptavidin (11205D, Invitrogen, California, USA). The mixture was then rotated at room temperature for 1 h. The supernatant was magnetically separated and discarded. Wash the samples with PBS for 5 times. The samples were diluted with a double loading buffer and heated at 100 °C for 10 min to elute the protein from the streptavidin beads. The samples were subjected to western blot.

### Cellular Thermal Shift Assay

After treatment with DMSO or G‐749 (5 µm) for 12 h, an equal number of cells were collected and each cell suspension was distributed into six PCR tubes, with 100 µL per tube. The proteins were heated in a metallic bath at the specified temperatures (45, 50, 55, 60, 65, and 70 °C) for 3 min and then cooled at room temperature for another 3 min. Then, the cells were freeze‐thawed using liquid nitrogen three times to lyse them, followed by centrifugation to collect the supernatant. These protein samples were prepared with 5× loading buffer and boiled. Changes in the amount of NAT10 directly bound to G‐749 were analyzed by Western blot.

### Molecular Docking

The 3D structures of G‐749 (78357765) were obtained from PubChem (https://pubchem.ncbi.nlm.nih.gov). The nuclear magnetic resonance (NMR) structures file of NAT10 (Q9H0A0) were downloaded from the UniProt database (https://www.uniprot.org). AutodockTools was used for hydrogenation, charge checking, specifying the atomic type as AD4, calculating the Gasteiger, and constructing the docking grid box for the protein structure. After Vina docking, the scores of the NAT10 protein and G‐749 docking combination were calculated. Force analysis and visualization were conducted using PyMOL software.

### Co‐Immunoprecipitation

Cell lysates were collected, and the total protein was quantified as mentioned above. After preclearing with normal IgG and beads (Santa Cruz Biotechnology), specific primary antibodies were added to 1 mg of total protein and incubated overnight on a rotator at 4 °C. Subsequently, the beads were added and rotated for another 6 h. After washing, the immunoprecipitation complex was analyzed by Western blot, and total cell lysates were used as input.

### Protein Purification and GST Pulldown

Full‐length NAT10 was cloned into the prokaryotic expression vector (pGEX‐4T‐1). Plasmids were transformed into *Escherichia coli* (BL21). Protein expression was induced with 1 mm isopropyl‐β‐D‐1‐thiogalactopyranoside (IPTG) after appropriate amplification. The collected bacteria pellet was lysed with lysozyme and ultrasonic crushing. The recombinant protein was purified using Glutathione Sepharose 4B beads (Cytiva) and an affinity chromatography column. The in vitro pulldown reaction containing 10 µg of GST‐NAT10 protein, 1 mg of total protein from ESCC cells, and 5 µm G‐749 was incubated overnight and the beads were washed. Finally, the bead‐bound complexes were analyzed by Western blot using indicated antibodies. 10% of the total cell lysate was used as input.

### In Vivo Experimental Metastasis Assay

KYSE150Luc‐LM5 cells expressing luciferase were intravenously injected into 6‐week‐old NCG mice to establish a lung metastasis model. Approximately 3–4 weeks after the G‐749 (5 mg kg^−1^) treatment, mice were intraperitoneally injected with substrate (D‐Luciferin, 150 mg kg^−1^) under anesthesia. The frequency of G‐749 administration was three times a week for three consecutive weeks. Tumor metastasis was monitored using the IVIS 100 imaging system (Caliper Life Sciences, Waltham, MA, USA). The lungs were dissected to observe the metastatic nodules, while the spleen, liver, and kidney were dissected to evaluate drug toxicity. The whole blood and serum from mice were collected for blood cell analysis and biochemical analysis, respectively.

### H&E Staining

The lung, spleen, liver, and kidney tissues were fixed with 10% paraformaldehyde solution, dehydrated, and embedded in paraffin. Subsequently, paraffin sections with a thickness of 5 µm were prepared and then baked in the oven for 2 h. After deparaffinizing and rehydrating, the sections were stained successively with Hematoxylin and Eosin. The images were captured using a microscope.

### Statistical Analysis

All statistical graphs were plotted by GraphPad Prism 10.0 software (La Jolla, CA, USA). Statistical analyses were evaluated using the unpaired 2‐tailed Student's t‐test and one‐way analysis of variance (ANOVA) with evaluation performed using Statistical Package for the Social Sciences (SPSS, Chicago, IL) software. Data are presented as the mean ± standard deviation (SD). The *P*‐value was utilized to assess differences, categorized as *P* < 0.05, *P* < 0.01, and *P* < 0.001.

### Ethics Approval

The animal experiment was approved by the Ethics Committee for Animal Experiments of Guangzhou Medical University, and the mice were cared for under standard conditions according to institutional guidelines.

## Conflict of Interest

The authors declare no conflict of interest.

## Author Contributions

Data curation: Y.J.Z., C.M.D., L.T., S.J.L., T.Y.X.; Conceptualization: Y.J.Z., B.L., W.W.X.; Validation: Y.J.Z., C.M.D., L.T., A.K.L., X.Y.S., Z.Y.P., M.L.H., L.X.; Writing‐original draft: Y.J.Z., W.W.X.; Software: C.M.D., S.J.L., X.W., X.Y.Z., C.S., S.L.; Visualization: C.M.D., T.Y.X., X.W., L.D., Z.M.O., R.Z., X.Y.S., S.L.; Investigation: C.M.D., L.T., Methodology: S.J.L., T.Y.X., L.D., C.S., A.K.L., W.D., M.L.H., C.Z.; Formal analysis: W.X., X.Y.Z, C.C.Z., J.L., X.B.C.; Funding acquisition: C.C.Z., R.Z., X.B.C., C.Z., B.L., W.W.X.; Project administration: C.C.Z, X.B.C.; Resource: L.D., C.S., Z.M.O., J.L., X.L.; Supervision: X.B.C., C.Z., B.L., W.W.X.; Writing‐review & editing: B.L., W.W.X.

## Supporting information



Supporting Information

Supplemental Tables

Supplemental Table 3

## Data Availability

The data that support the findings of this study are available from the corresponding author upon reasonable request.
