# Supplementary material for: NAT10 Increases Lysosomal Acidification to Promote Esophageal Cancer Metastasis via ac4C Acetylation of ATP6V0E1 mRNA
Source: Adv Sci (Weinh). 2025 Jul 29;12(31):e02931. doi: 10.1002/advs.202502931 (PMC12376557; doi:10.1002/advs.202502931)
Supplement: Supplementary file 1 — Supporting Information [file ADVS-12-e02931-s003.pdf]

ADVANCED SCIENCE
Open Access

Supporting Information

for *Adv. Sci.*, DOI 10.1002/advs.202502931

NAT10 Increases Lysosomal Acidification to Promote Esophageal Cancer Metastasis via ac4C Acetylation of ATP6V0E1 mRNA

Yu-Juan Zhan, Chun-Miao Deng, Lin Tang, Shu-Jun Li, Tao-Yang Xu, Xian Wei, Xin-Yi Zhang, Can-Can Zheng, Li Deng, Cui Shao, Zhong-Min Ouyang, Alfred King-Yin Lam, Rong Zhang, Jun Liu, Xing-Yuan Shi, Zhen-Yu Pan, Wei Dai, Ming-Liang He, Simon Law, Xu Li, Xiao-Bing Chen, Cheng Zhou*, Bin Li* and Wen-Wen Xu**

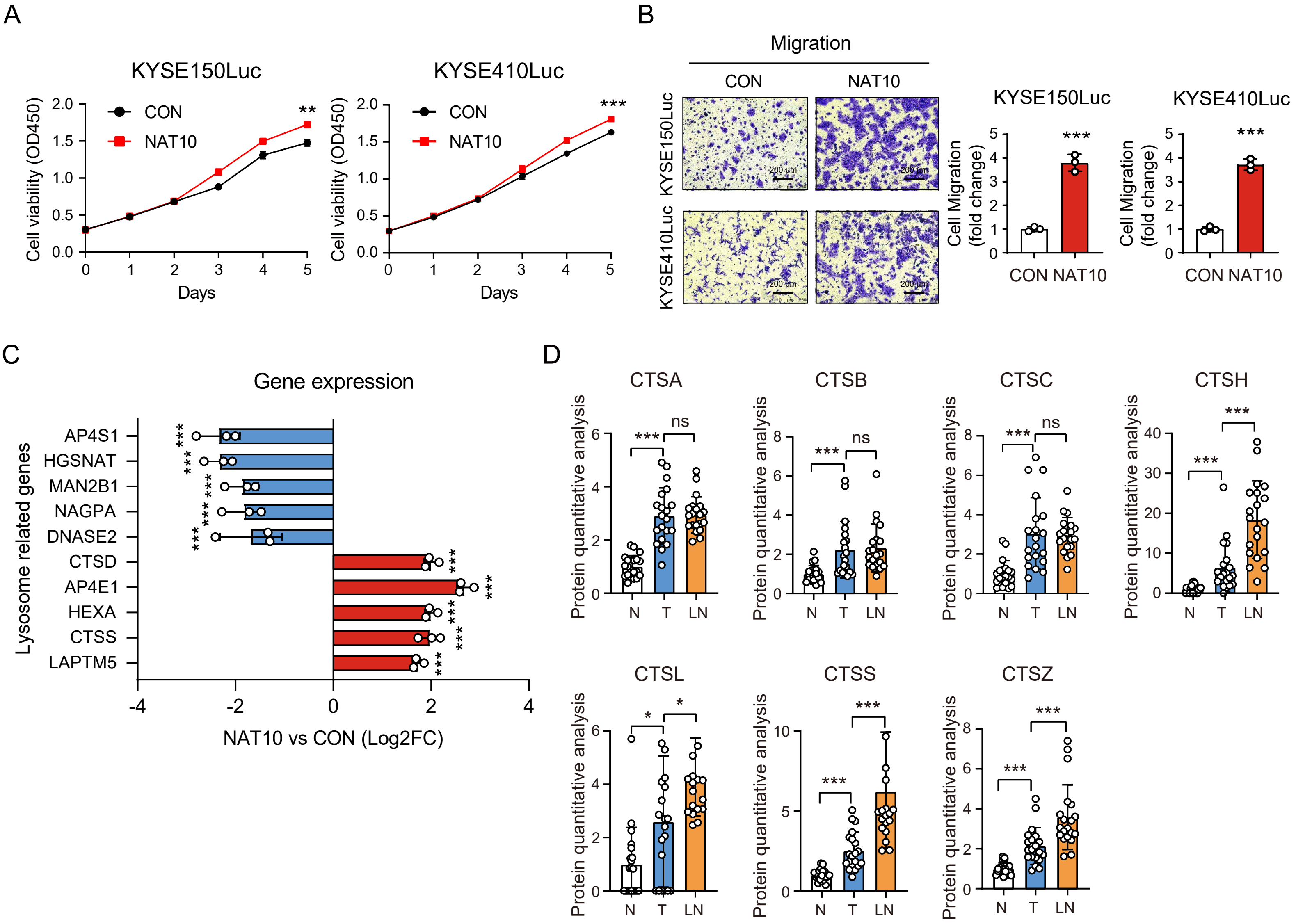

Supplementary Fig. 1 The effects of NAT10 on cancer cell proliferation, migration, and lysosome-related genes. (A-B) The effects of NAT10 on cancer cell proliferation at the corresponding time and migration at 36 hours. KYSE150Luc and KYSE410Luc cells overexpressing NAT10 were subjected to cell proliferation (A) and migration (B) assays. Scale bar: 200 μm. **(C)** mRNA levels of lysosome-related genes in NAT10-overexpressing cells were detected by RT-qPCR. **(D)** The expression of cathepsin (CTS) A/B/C/H/L/S/Z in proteomic data. Bars, SDs; ns, no significance; * $P < 0.05$; ** $P < 0.01$; *** $P < 0.001$. N, Normal; T, Tumor; LN, Lymphatic metastasis.

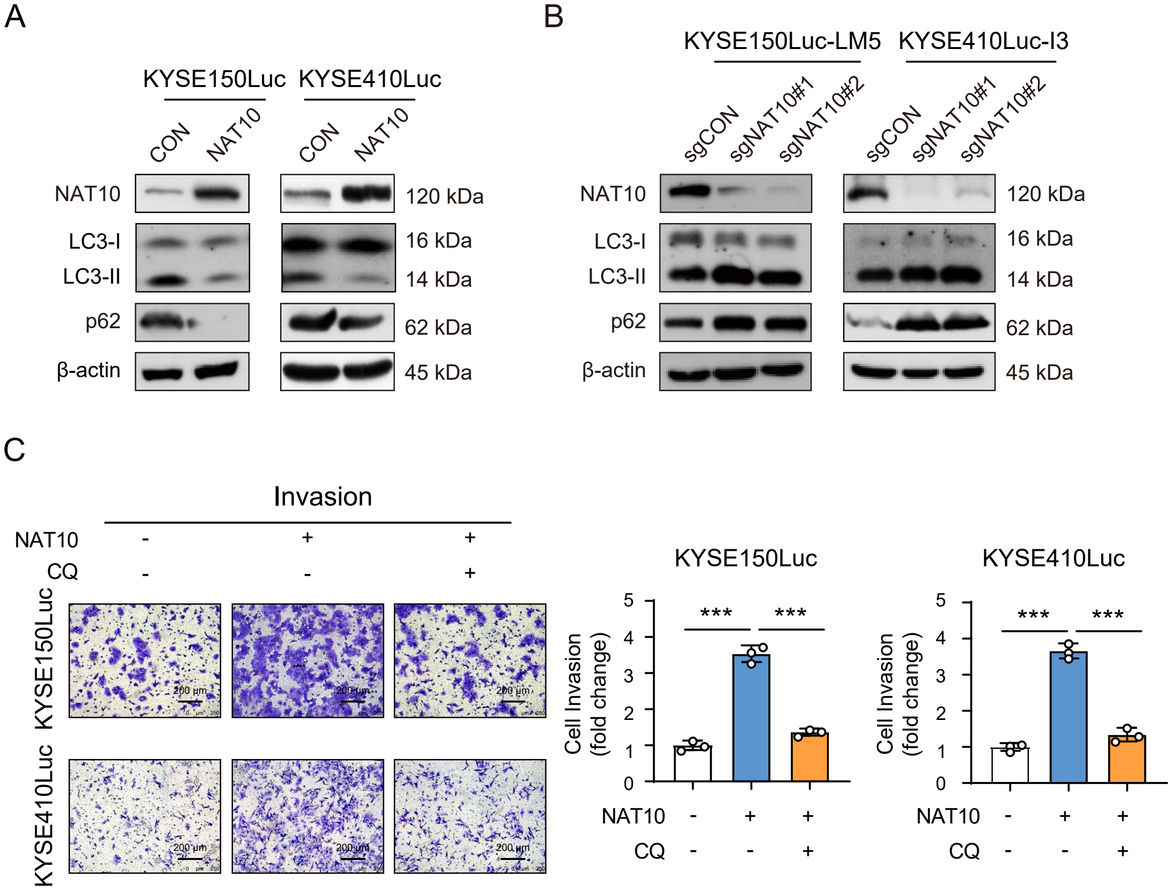

Supplementary Fig. 2 NAT10 regulates aytophagy and lysosomal pathway. (A) Western blot analysis detecting the protein expression of LC3, p62, and NAT10 in NAT10-overexpressing or control cells. **(B)** Monitoring the expression levels of LC3, p62, and NAT10 in NAT10-knockout or control cells. **(C)** Blockade of autophagy attenuated the effect of NAT10 on ESCC invasion. KYSE150Luc and KYSE410Luc cells with NAT10 overexpression were treated with chloroquine (CQ, 10 μM), then subjected to transwell assay. Scale bar: 200 μm. Bars, SDs; *** $P < 0.001$.

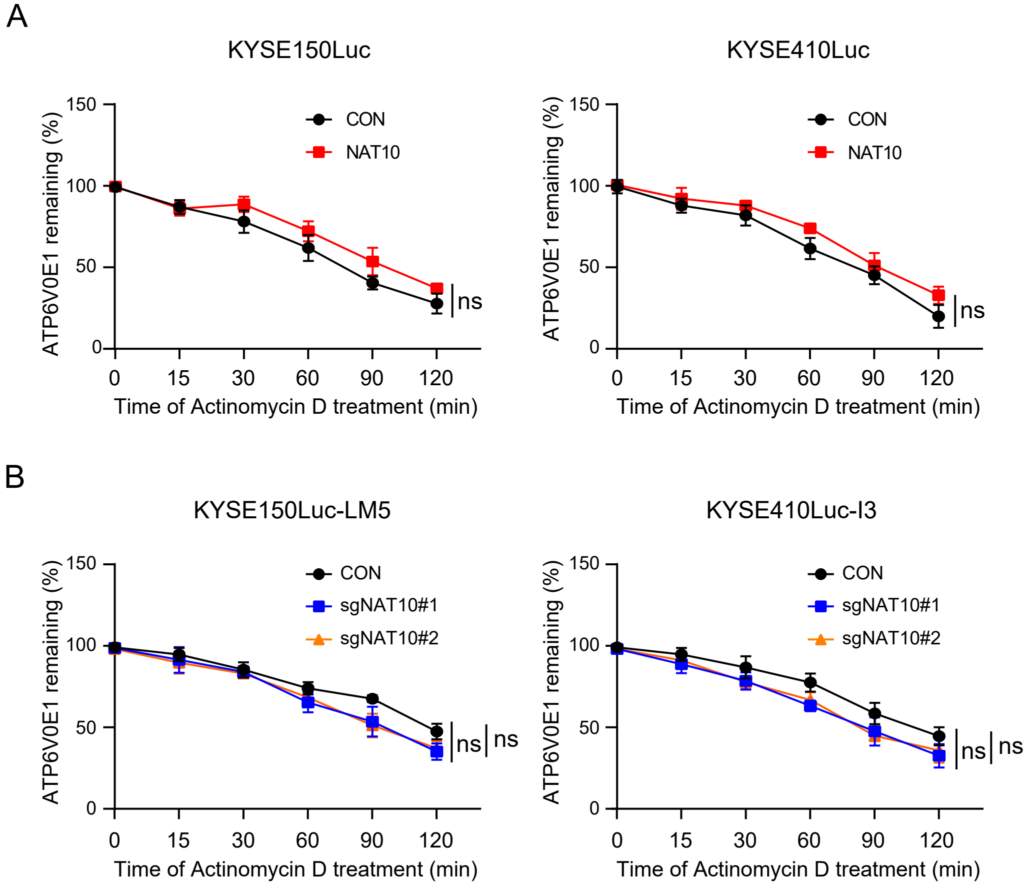

Supplementary Fig. 3 The effects of NAT10 on the mRNA stability of ATP6V0E1. (A-B) The degradation of ATP6V0E1 mRNA was monitored using the mRNA half-life assay in KYSE150Luc and KYSE410Luc cells with NAT10-overexpression (A), as well as in KYSE150Luc-LM5 and KYSE410Luc-I3 cells with NAT10 knockout (B). Bars, SDs; ns, no significance.

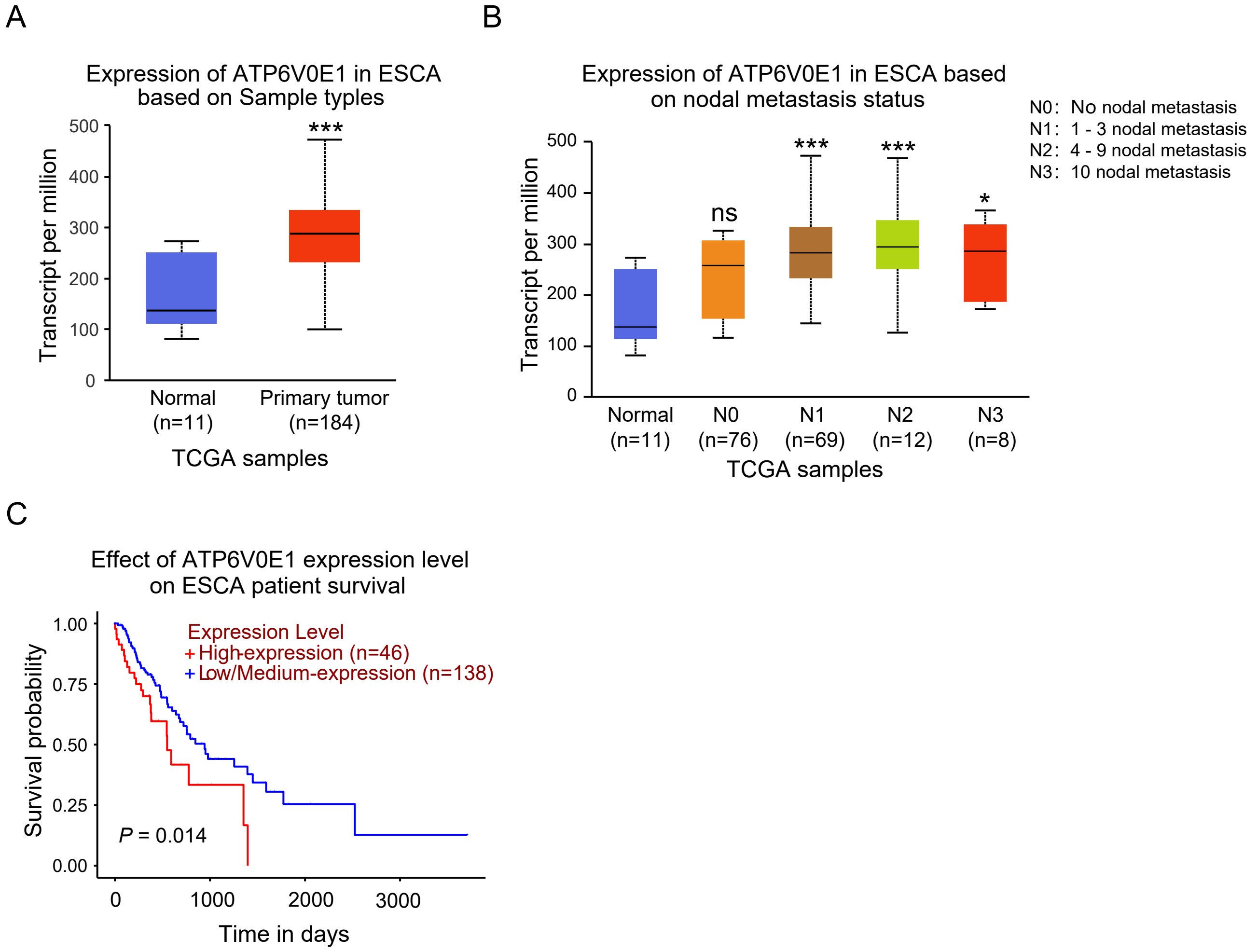

Supplementary Fig. 4 The clinical significance of ATP6V0E1 in esophageal cancer progression. (A) The expression of ATP6V0E1 in the cohort of esophageal carcinoma in TCGA database. **(B)** Analysis of the ATP6V0E1 expression in esophageal cancer patients with different nodal metastasis status on UALCAN website. **(C)** Survival analysis of patients with esophageal cancer in TCGA database stratified based on ATP6V0E1 expression level. Bars, SDs; ns, no significance; *, $P < 0.05$; ***, $P < 0.001$.

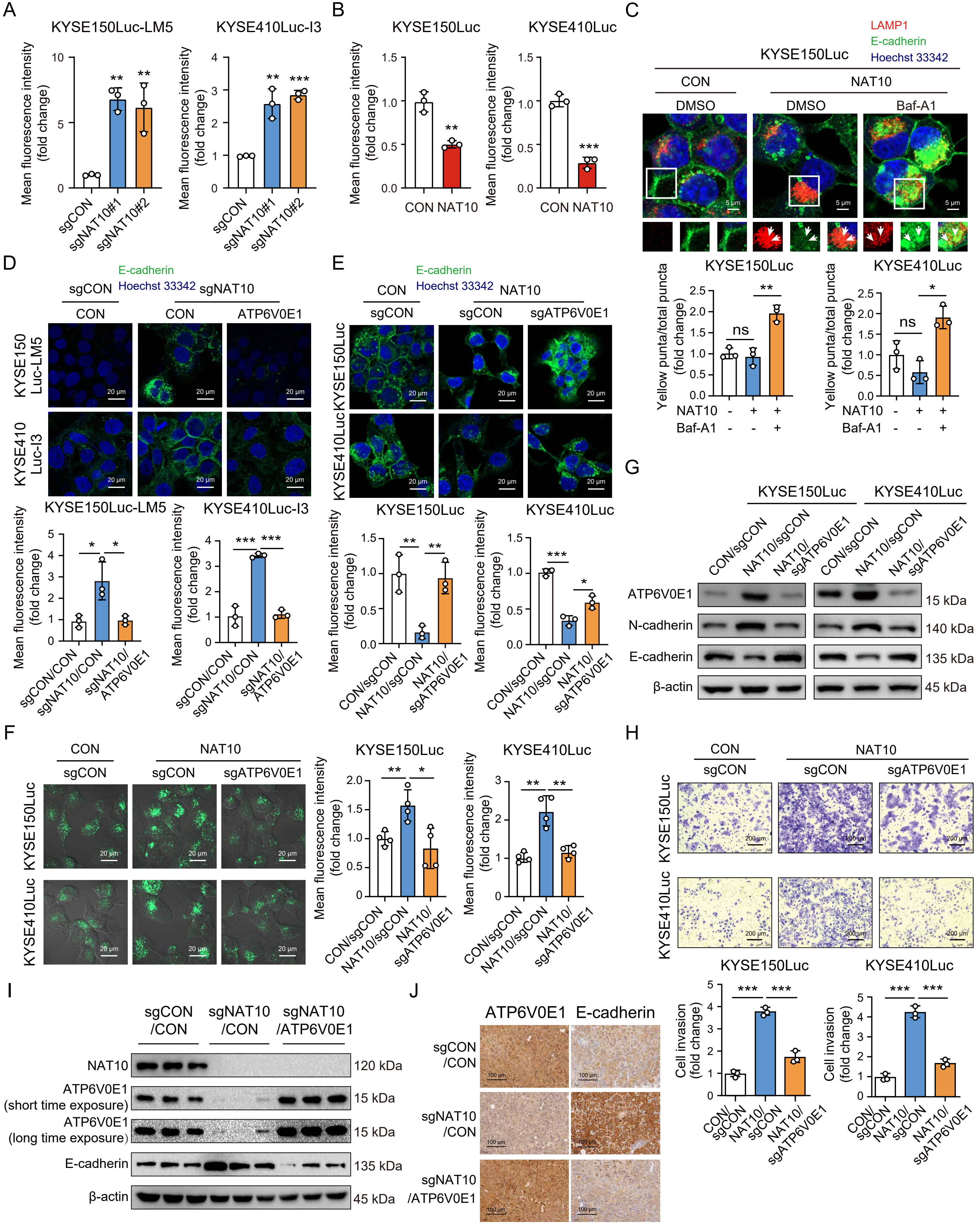

Supplementary Figure 5

Supplementary Fig. 5 NAT10 accelerates the lysosomal degradation of E-cadherin via regulating the ATP6V0E1 expression to promote cancer metastasis. (A-B) The fluorescence intensity of membrane localization of E-cadherin in ESCC cells. **(C)** The co-localization of E-cadherin with LAMP1 was detected using confocal microscopy in control or NAT10-overexpressing cells in the presence or absence of Baf-A1. The white arrows indicate the co-localization of E-cadherin and LAMP1. Scale bar: 5 μm. **(D-E)** Immunofluorescence showing ATP6V0E1 mediated the effect of NAT10 on the expression of E-cadherin. Scale bar: 20 μm. **(F)** LysoTracker-Green staining showing that ATP6V0E1 mediated the effect of NAT10 on lysosomal acidification. Scale bar: 20 μm. **(G-H)** Western blot analysis (G) and the Boyden chamber assay (H) indicated that ATP6V0E1 knockout attenuated the invasion and EMT phenotypes triggered by NAT10 overexpression in cancer cells. Scale bar: 200 μm. **(I-J)** Detection of E-cadherin and ATP6V0E1 by Western blot (I) and IHC (J) assays in lung metastatic tissue from the tail vein metastasis model. Scale bar: 100 μm. Bars, SDs; * $P < 0.05$; ** $P < 0.01$; *** $P < 0.001$.

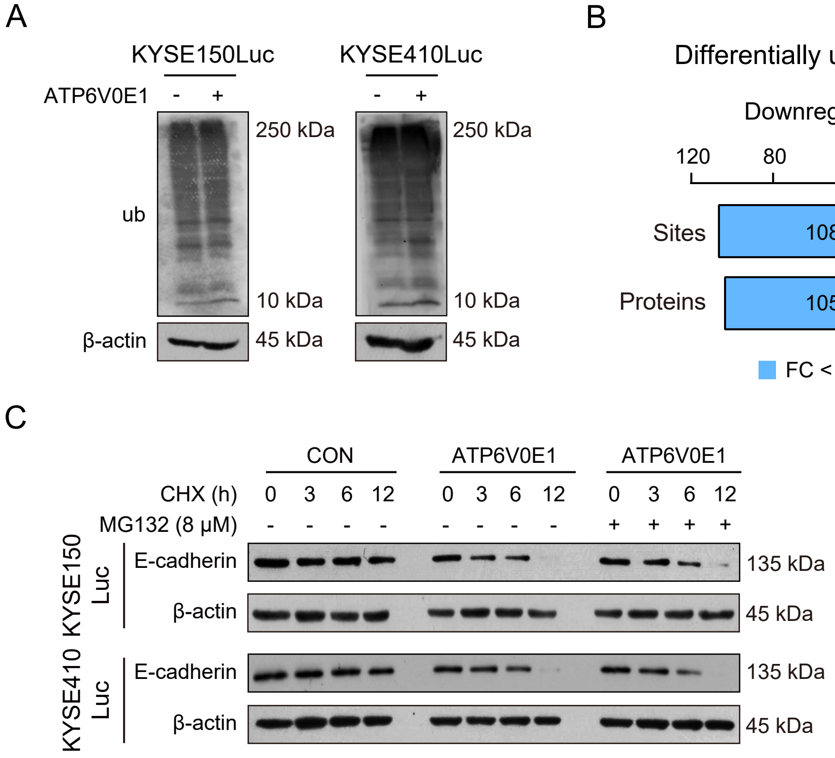

Supplementary Fig. 6 The effects on ubiquitin-dependent degradation by ATP6V0E1. (A) The total level of ubiquitinated protein was detected using Western blot analysis after ATP6V0E1-overexpression. **(B)** The proteomic analysis of ubiquitination modifications identified upregulated (red) and downregulated (blue) ubiquitination sites and proteins following the overexpression of ATP6V0E1, based on significant changes in abundance. **(C)** In the presence of MG132 (8 μM) or CHX (50 μg/mL), the expression of E-cadherin was shown in ATP6V0E1-overexpressing cells or control.

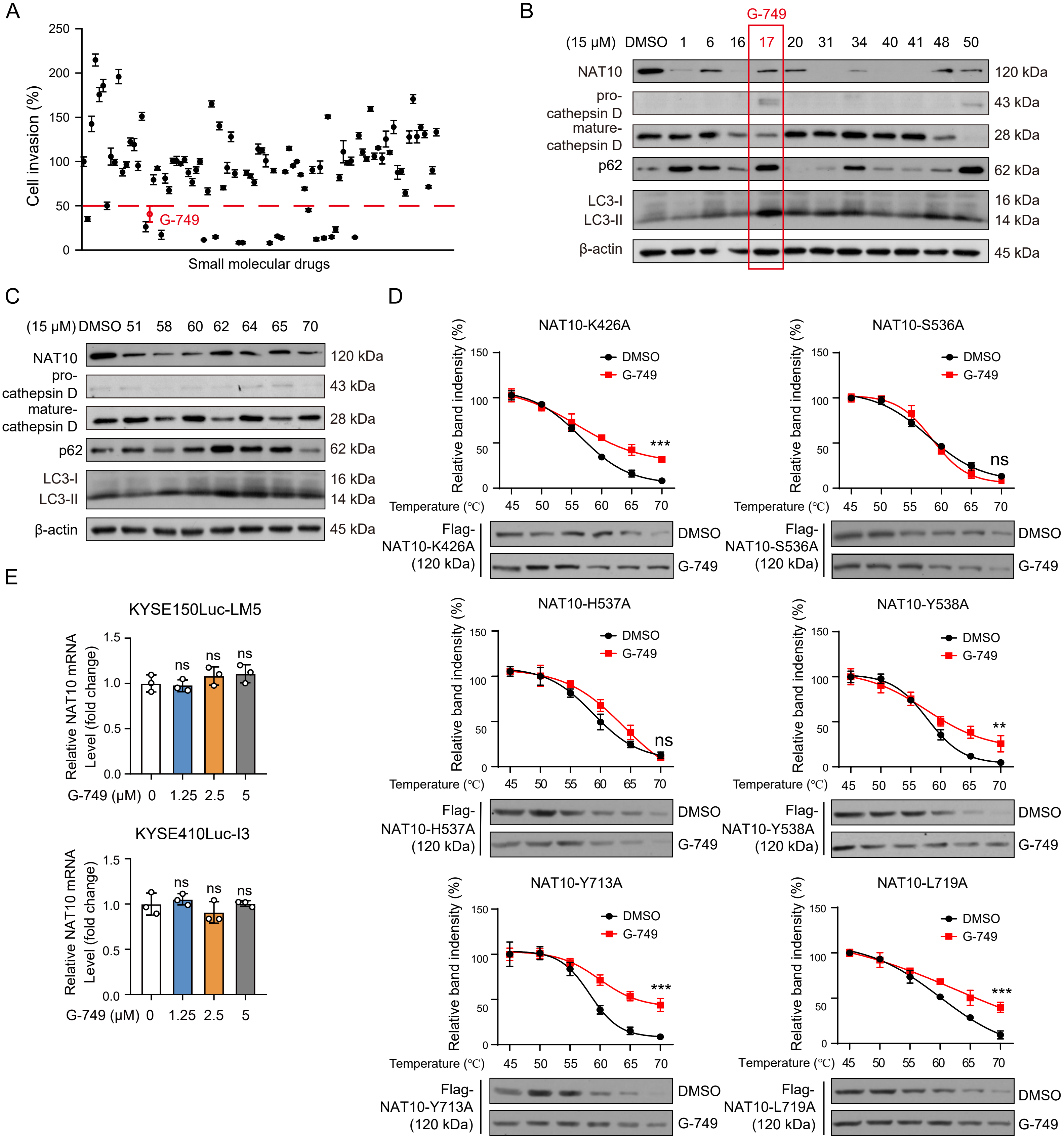
A
Cell invasion (%)
Small molecular drugs
G-749
B
(15 μM) DMSO 1 6 16 17 20 31 34 40 41 48 50
G-749
NAT10
pro-cathepsin D
mature-cathepsin D
p62
LC3-I
LC3-II
β-actin
120 kDa
43 kDa
28 kDa
62 kDa
16 kDa
14 kDa
45 kDa
C
(15 μM) DMSO 51 58 60 62 64 65 70
D
NAT10-K426A
NAT10-S536A
NAT10-H537A
NAT10-Y538A
NAT10-Y713A
NAT10-L719A
Relative band indensity (%)
Temperature (℃)
DMSO
G-749
***
**
ns
Flag-NAT10-K426A (120 kDa)
Flag-NAT10-S536A (120 kDa)
Flag-NAT10-H537A (120 kDa)
Flag-NAT10-Y538A (120 kDa)
Flag-NAT10-Y713A (120 kDa)
Flag-NAT10-L719A (120 kDa)
E
KYSE150Luc-LM5
KYSE410Luc-I3
Relative NAT10 mRNA Level (fold change)
G-749 (μM) 0 1.25 2.5 5
ns

Supplementary Fig. 7 G-749 is screened as an inhibitor of ESCC metastasis by suppressing lysosomal acidification. (A) Quantification of the effect of 91 compounds on ESCC cells invasion by invasion chamber assay. **(B-C)** The expression of NAT10, cathepsin D, LC3, and p62 in ESCC cells treated with 18 candidate compounds was detected using Western blot analysis. **(D)** CETSA analysis indicating the specific binding sites between G-749 and NAT10. **(E)** RT-qPCR detection of NAT10 mRNA levels in KYSE150Luc-LM5 and KYSE410Luc-I3 cells treated with G-749 at different concentrations. Bars, SDs; ns, no significance; ** $P < 0.01$; *** $P < 0.001$.

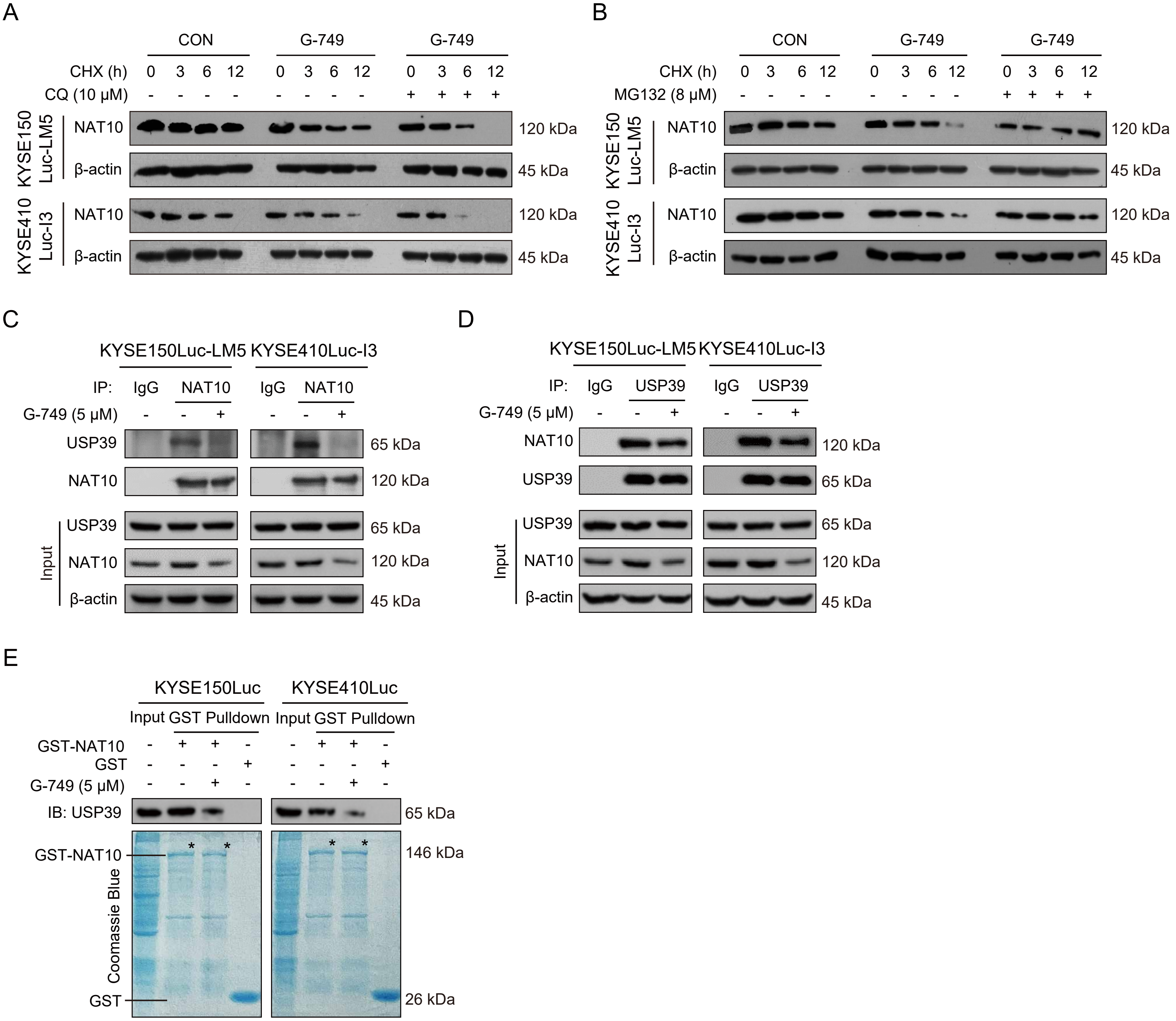

Supplementary Fig. 8 G-749 disrupts USP39-NAT10 interaction. (A-B) Detection of NAT10 by Western blot assay. KYSE150Luc-LM5 and KYSE410Luc-I3 cells were pre-treated with CQ (10 μM) or MG132 (8 μM) and then treated with G-749 (5 μM) for 12 hours, during which cells were treated with CHX (50 μg/mL) in a time gradient (0, 3, 6, 12 hours). The protein level was examined by Western blot. **(C-D)** Co-IP results showing G-749 weakens the endogenous NAT10-USP39 interaction in KYSE150Luc-LM5 or KYSE410Luc-I3 cells. Normal IgG was used as a negative control. **(E)** GST-NAT10 pulldown of USP39 was conducted in KYSE150Luc or KYSE410Luc cells after *in vitro* incubation with or without G-749. GST was used as a negative control.

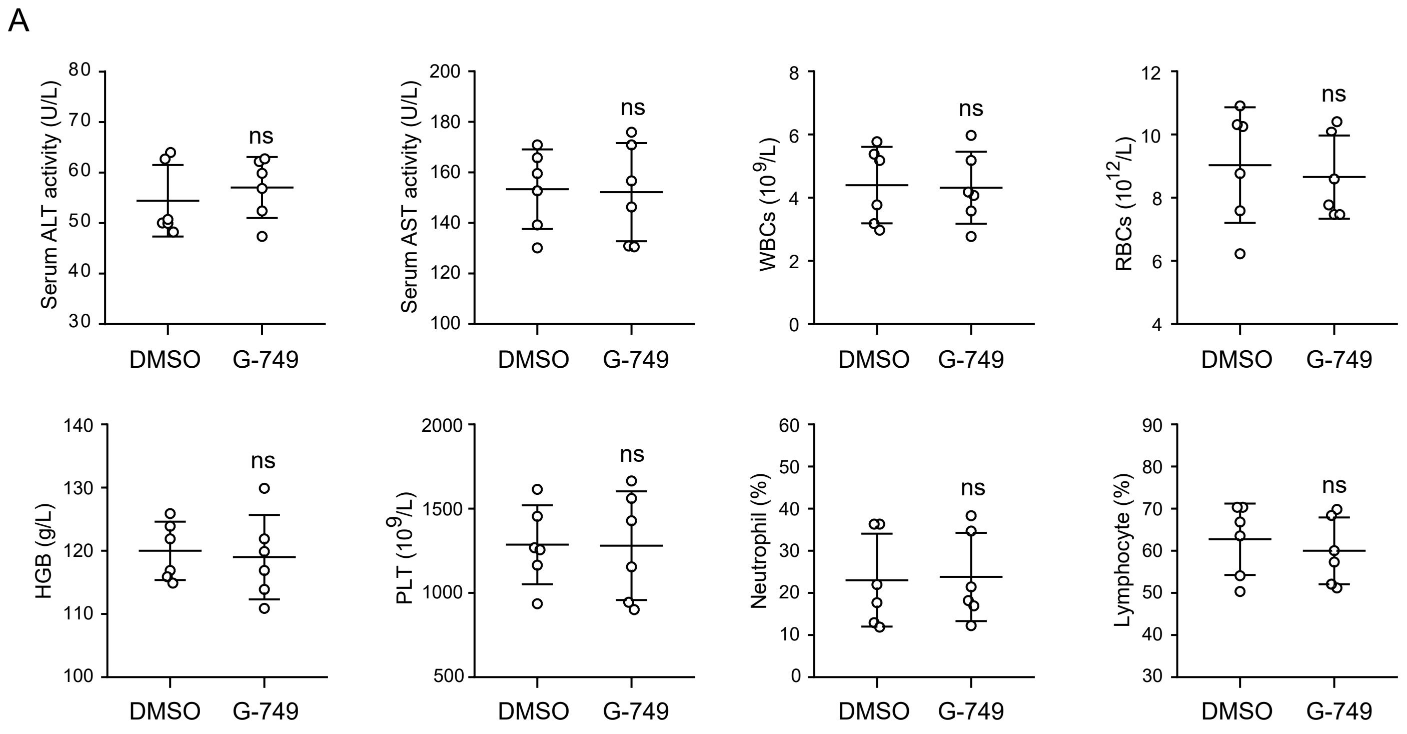

Supplementary Fig. 9 Blood chemistry analysis. (A) Comparison of ALT, AST, WBCs, RBCs, HGB, PLTs, neutrophil, and lymphocyte levels in the mice treated with G-749 or control. Bars, SD; ns, no significance.

A

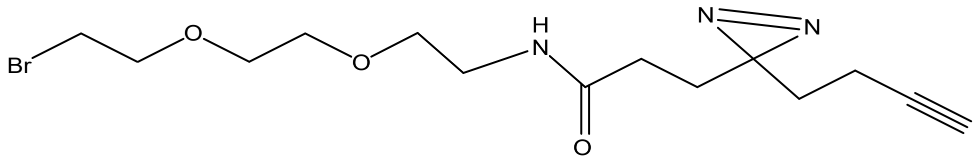

control probe (CP)

Chemial Formula: $C_{14}H_{22}BrN_3O_3$

B

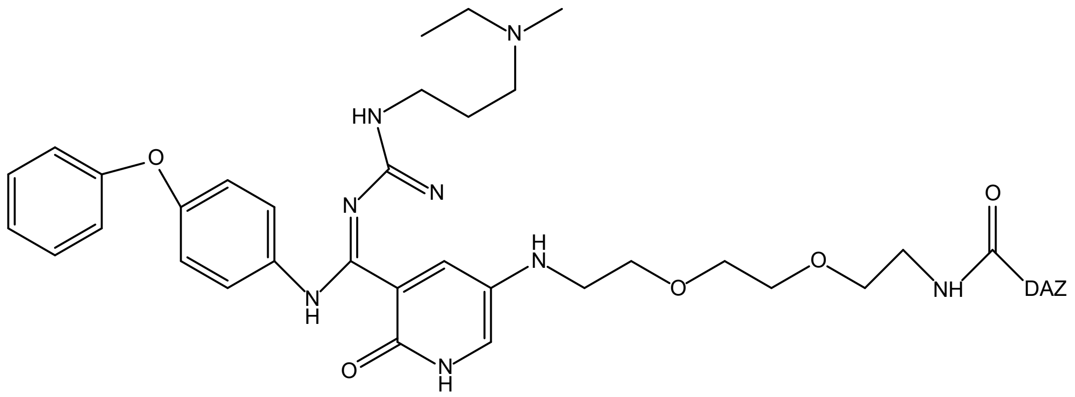

G-749 probe (GP)

Chemial Formula: $C_{39}H_{46}BrN_9O_5$

Supplementary Fig. 10 Structure of the diazirine photo–cross-linking group without [control probe (CP), A] or with [G-749 probe (GP), B] G-749.
