# Supplementary material for: NAT10 Increases Lysosomal Acidification to Promote Esophageal Cancer Metastasis via ac4C Acetylation of ATP6V0E1 mRNA
Source: Adv Sci (Weinh). 2025 Jul 29;12(31):e02931. doi: 10.1002/advs.202502931 (PMC12376557; doi:10.1002/advs.202502931)
Supplement: Supplementary file 2 — Supplemental Tables [file ADVS-12-e02931-s001.pdf]

ADVANCED SCIENCE
Open Access

Supporting Information

for *Adv. Sci.*, DOI 10.1002/advs.202502931

NAT10 Increases Lysosomal Acidification to Promote Esophageal Cancer Metastasis via ac4C Acetylation of ATP6V0E1 mRNA

Yu-Juan Zhan, Chun-Miao Deng, Lin Tang, Shu-Jun Li, Tao-Yang Xu, Xian Wei, Xin-Yi Zhang, Can-Can Zheng, Li Deng, Cui Shao, Zhong-Min Ouyang, Alfred King-Yin Lam, Rong Zhang, Jun Liu, Xing-Yuan Shi, Zhen-Yu Pan, Wei Dai, Ming-Liang He, Simon Law, Xu Li, Xiao-Bing Chen, Cheng Zhou*, Bin Li* and Wen-Wen Xu**

Supplementary Table 1. Genes of v-ATPase subunits enriched in Humans.

Subunit Designation	Human Gene
**V1 Subunits**	
A	ATP6V1A
B	ATP6V1B1
	ATP6V1B2
C	ATP6V1C1
	ATP6V1C2
	ATP6V1C3
D	ATP6V1D
E	ATP6V1E1
	ATP6V1E2
F	ATP6V1F
G	ATP6V1G1
	ATP6V1G2
	ATP6V1G3
H	ATP6V1H
**V0 Subunits**	
a	ATP6V0A1
	ATP6V0A2
	TCIRG1
	ATP6V0A4
c	ATP6V0C
c’	-
c’’	ATP6V0B
d	ATP6V0D1
	ATP6V0D2
e	ATP6V0E1
	ATP6V0E2

f	RNASEK
Ac45	ATP6AP1
M8-9	ATP6AP2
**Chaperone**	
VMA12	TMEM199
	VMA21
	CCDC115

Supplementary Table 2. Correlation between ATP6V0E1 expression levels and clinicopathological parameters in 211 patients with esophageal cancer.

Variable	n	Low ATP6V0E1	High ATP6V0E1	*P* value
Age (years)				
≤55	28	12	16	
>55	183	70	113	0.64
Gender				
Female	44	21	23	
Male	167	61	106	0.18
T stage				
1/2	50	23	27	
3/4	161	59	102	0.26
N stage				
N0	101	47	54	
N1/N2/N3	110	12	75	**0.03***
Grade				
I & II	163	70	93	
III & IV	48	12	36	**0.02***

Abbreviations: T, tumor invasion depth; N, lymph node involvement.

$P < 0.05$ was considered significant. The bold type represents *P* values smaller than 0.05.

Supplementary Table 3. (separate file)

Cell ubiquitination-proteome dataset

Supplementary Table 4. The list of 91 Small molecular and their cell invasion rates on KYSE150Luc-LM5 cells.

No.	Small molecular	Cell invasion rate (%)
1	LMK-235	35.2
2	GNE9605	142.6
3	LDN-57444	214.8
4	PFI-4	175.8
5	GSK 5959	185.8
6	MHY1485	50
7	BYL-719(Alpelisib)	105.7
8	DBEQ	99.3
9	AZ20	196.0
10	CZC24832	88.0
11	GNE7915	96.6
12	JNJ-26854165 (Serdemetan)	122.5
13	Bioymifi	119.2
14	kb NB 142-70	95.2
15	PIK-93	151.2
16	DCC-2036 (Rebastinib)	26.4
17	G-749	40.8
18	Gandotinib (LY2784544)	79.6
19	SGI-1776 free base	92.6
20	CH5132799	17.4
21	SNS-314 Mesylate	81.2
22	PR-619	67.7
23	CID-2011756	100.9
24	Tenovin-3	100.7
25	Methylisoindigotin	97.6
26	BIX02189	102.3
27	BAM7	87.6
28	PF-06447475	76.9
29	CID755673	90.4
30	Roxadustat (FG-4592)	100.2
31	PP121	11.6
32	MK8745	66.5
33	NSC319726	165.2
34	PIK-90	14.8
35	Torkinib (PP242)	140.3
36	P22077	70.5

No.	Small molecular	Cell invasion rate (%)
37	MC1568	93.0
38	MS436	128.1
39	ML323	86.6
40	GDC-0032	8.5
41	CZC-54252	8.3
42	Theophylline monohydrate	87.5
43	Bengenin	83.9
44	VGX-1027	76.5
45	kb-NB77-78	114.3
46	IOX 2	112.7
47	CX-6258	101.0
48	NMS 873	7.9
49	Moclobemide	89.8
50	Sodium Aescinate	15.7
51	Methyl protocatechuate	13.8
52	(20S)-Protopanaxadiol	88.7
53	Histone Deacetylase Inhibitor III	115.0
54	SMI-4a	95.4
55	TAK901	100.2
56	TCS PIM-1 1	85.4
57	LRRK2-IN-1	569.4
58	GSK2578215A	45.1
59	ETP46464	90.6
60	GSK-1059615	12.2
61	CEP-32496	93.7
62	XL147 analogue	13.6
63	XMD17-109	150.6
64	GSK-2636771	14.8
65	BAY 87-2243	23.4
66	TG100-115	81.2
67	Y-27632 dihydrochloride	111.2
68	Lacidipine	98.7
69	Nilvadipine	100.6
70	Tetracaine hydrochloride	14.4
71	Loganin	129.4
72	L-Adrenaline	110.8
73	Clevidipine butyrate	102.8
74	Tiplaxtinin(PAI-039)	159.6

No.	Small molecular	Cell invasion rate (%)
75	Manidipine dihydrochloride	106.0
76	ISRIB	115.5
77	Azelnidipine	103.6
78	Sulfacetamide sodium	125.6
79	Cilnidipine	110.2
80	Dexibuprofen	139.0
81	BML-210	87.8
82	UF010	89.1
83	PTACH	64.8
84	Pimelic diphenylamide 106	127.8
85	Manidipine	170.7
86	RG2833 (RGFP109)	128.2
87	GNE-0877	138.6
88	SHP099 hydrochloride	131.1
89	CZC-25146	71.6
90	SMER28	90.1
91	ACY-241	133.3

Supplementary Table 5. Oligonucleotides sequences in this study.

**Primers used for the qRT-PCR assay**	
hGAPDH-F	CCATCACCATCTTCCAGGAG
hGAPDH-R	ATGATGACCCTTTTGGCTCC
hNAT10-F	GGAGTAGCTGAGCGGCAAA
hNAT10-R	CTTCTGCAGCTGTCGCATTC
**Primers used for the acRIP-qRT-PCR assay**	
ATP6V0E1-F	AGCCATCTCAACAGAATCGCA
ATP6V0E1-R	ACAAACACCGAGCCCAGATT
**Primers used for luciferase reporter assay**	
R-luc.qPCR-F	GGGCGAGAAAATGGTGCTTG
R-luc.qPCR-R	GAATGGCTCCAGGTAGGCAG
F-luc.qPCR-F	GCTCAGCAAGGAGGTAGGTG
F-luc.qPCR-R	TGATCAGAATGGCGCTGGTT
**Primers used for sgRNA**	
ATP6V0E1#1-F1	AGTGAGGCCGTGATACGCCA GTTTTAGAGCTAGAAATAGC
ATP6V0E1#1-R1	TGGCGTATCACGGCCTCACT CGGTGTTTCGTCCTTTCCAC
ATP6V0E1#2-F2	GAGATAGCAGCAAACTGAAC GTTTTAGAGCTAGAAATAGC
ATP6V0E1#2-R2	GTTCAGTTTGCTGCTATCTC CGGTGTTTCGTCCTTTCCAC
